# Bufei Yishen Formula Restores Th17/Treg Balance and Attenuates Chronic Obstructive Pulmonary Disease *via* Activation of the Adenosine 2a Receptor

**DOI:** 10.3389/fphar.2020.01212

**Published:** 2020-08-07

**Authors:** Peng Zhao, Xuefang Liu, Haoran Dong, Yange Tian, Suxiang Feng, Di Zhao, Zhouxin Ren, Lanxi Zhang, Jiansheng Li

**Affiliations:** ^1^ Co-construction Collaborative Innovation Center for Chinese Medicine and Respiratory Diseases by Henan & Education Ministry of P.R. China, Henan University of Chinese Medicine, Zhengzhou, China; ^2^ Henan Key Laboratory of Chinese Medicine for Respiratory Disease, Henan University of Chinese Medicine, Zhengzhou, China; ^3^ Academy of Chinese Medical Sciences, Henan University of Chinese Medicine, Zhengzhou, China

**Keywords:** Bufei Yishen formula, chronic obstructive pulmonary disease, adenosine 2a receptor, Th17 cell, Treg cell, Th17/Treg balance

## Abstract

Bufei Yishen formula (BYF) is a Traditional Chinese Medicine (TCM) reported to ameliorate chronic obstructive pulmonary disease (COPD) by regulating the balance between T helper (Th) 17 and regulatory T (Treg) cells. However, its mechanism remains unknown. Therefore, this study aimed to explore the underlying mechanisms of BYF. Naïve CD4+ T cells were exposed to anti-CD3, anti-CD28, transforming growth factor (TGF)-β, and/or interleukin (IL)-6 to promote their differentiation into Th17 or Treg cells. A rat model of cigarette smoke- and bacterial infection-induced COPD was established and orally treated with BYF and/or an adenosine 2a receptor (A2aR) antagonist. Then, the rats were sacrificed, their lung tissues were removed for histological analysis, and their spleens were collected to evaluate Th17 and Treg cells. The results showed that BYF significantly suppressed Th17 cell differentiation and its related cytokines and enhanced Treg cell differentiation and its related cytokines. In addition, BYF activated the A2aR, increased the levels of p-signal transducer and activator of transcription (STAT)5, and decreased the level of p-STAT3 in Treg and Th17 cells. The A2aR antagonist suppressed the changes induced by BYF treatment in Th17 and Treg cells. Furthermore, the A2aR antagonist diminished the therapeutic effect of BYF on COPD, as indicated by the lung injury scores, bronchiole wall thickness, small pulmonary vessels wall thickness, bronchiole stenosis, alveolar diameters, decrease in inflammatory cytokines, increase in alveolar number, and lung functions. Similarly, the A2aR antagonist reversed the effects of BYF on the proportion of Th17 and Treg cells in the spleen. Additionally, BYF increased the protein and mRNA levels of A2aR and regulated the phosphorylation of STAT3 and STAT5 in spleen and lung tissues, which were inhibited by cotreatment with the A2aR antagonist. In conclusion, this study suggested that BYF exhibited its anti-COPD efficacy by restoring the Th17/Treg balance *via* activating A2aR, which may provide evidence for the clinical application of BYF in the treatment of COPD.

## Introduction

Chronic obstructive pulmonary disease (COPD) is a heterogeneous syndrome associated with abnormal inflammatory immune responses of the lung. COPD is a worldwide public health challenge because of its high prevalence and related disability and mortality (Wang et al., 2018; Maigeng et al., 2019). Although the precise pathogenesis of COPD remains unclear, Th17/Treg imbalance is considered to be an essential inducer of COPD development (Barnes, 2016). In COPD patients, the Th17/Treg cell balance shifts toward Th17 cells, which triggers inflammatory responses in the airways and lungs and exacerbates alveolar destruction by producing interleukin-17 (IL-17) (Brusselle et al., 2011; Eppert et al., 2013). For instance, Th17 expansion and higher IL-17A concentrations are found in the sputum of COPD patients and are tightly correlated with increased neutrophil chemotactic mediators and airway obstruction (Le Rouzic et al., 2017; Cervilha et al., 2019). *In vitro*, Th17 cells can be generated from naïve T-cells induced by transforming growth factor (TGF)-β1 and IL-6. Additionally, IL-17A produced by Th17 cells is tightly regulated by transcriptional factors, including signal transducer and activator of transcription 3 (STAT3) and retinoid-related orphan receptor (ROR)γt, which are activated by combinations of cytokines, including IL-6 and TGF-β (Jin et al., 2014; Cervilha et al., 2019). In contrast to Th17 cells, Treg cells have immunoregulatory functions and secrete anti-inflammatory cytokines to strengthen immune tolerance and inhibit inflammatory responses. Treg levels are decreased in COPD patients and mice induced by chronic cigarette smoke exposure (Yang et al., 2011; Zhang et al., 2016). For example, the number of Tregs in the small airways and peripheral blood of COPD patients is lower compared with healthy individuals. In addition, Treg cells in COPD patients exhibit a lower capacity to inhibit inflammation (Jin et al., 2014; Sales et al., 2017). Therefore, the Th17 and Treg balance is essential to maintain immune homeostasis and inhibit inflammation in COPD.

The adenosine 2a receptor (A2aR) is widely expressed on T cell subsets and may regulate cytokine production in activated T lymphocytes, suggesting that this receptor may play a role in immune homeostasis and inflammatory processes (Zarek et al., 2008; Li N. et al., 2012). A2aR stimulation promotes the generation of forkhead box p3 (Foxp3)+ regulatory T cells and reduces Th17-cell subtypes (Zarek et al., 2008). Additionally, A2aR-knockout mice sensitized and challenged with ragweed exhibit enhanced airway inflammation and hyperresponsiveness compared with wild-type mice (Haskó et al., 2008). Further, A2aR agonist treatment inhibits airway inflammation in allergen-sensitized and -challenged brown Norway rats (Bonneau et al., 2006; Trevethick et al., 2008). In addition, A2aR signaling pathways activate protein kinase A (PKA), resulting in CRE binding protein (CREB) phosphorylation, and A2aR silencing on T cells can block their differentiation toward Tregs by inhibiting PKA/CREB activation (Yang et al., 2015; Masjedi et al., 2019). Thus, activating A2aR to restore the Th17/Treg balance may offer a potential therapeutic strategy for COPD.

Bufei Yishen formula (BYF) (patent: ZL.201110117578.1) is a traditional Chinese formula composed of twelve Chinese medicines, including Ginseng Radix et Rhizoma, Astragali Radix, Corni Fructus, Lycii Fructus, Schisandrae Chinensis Fructus, Fritillariae Thunbergii Bulbus, Perillae Fructus, Citri Reticulatae Pericarpium, Epimedii Folium, Paeoniae Rubra Radix, Pheretima, and Ardisiae Japonicae Herba. Previous clinical studies reported the beneficial effects of BYF on measured outcomes in stable COPD patients over the 6-month treatment and 12-month follow-up periods. Specifically, BYF alleviated COPD symptoms by reducing the exacerbation frequency, delaying acute exacerbation, and improving pulmonary function and exercise capacity (Li S. et al., 2012). We previously conducted a system analysis by integrating transcriptomics, proteomics, metabolomics, and system pharmacology and showed that BYF achieved its beneficial effect on COPD by regulating immune responses, inflammatory responses, lipid metabolism, and other processes (Li et al., 2015; Li J. et al., 2016). Subsequently, we demonstrated that BYF restored the Th17/Treg balance in spleens and mesenteric lymph nodes and modulated the activities of STAT3 and STAT5 in COPD rats (P et al., 2018). However, the detailed mechanisms are still unknown and require further investigation.

The present work aimed to explore the mechanism by which BYF restores the Th17/Treg balance *in vitro* and *in vivo* and evaluate the critical role of A2aR in the subsequent anti-COPD effect of BYF.

## Materials and Methods

### Chemicals and Reagents

Tobacco was purchased from Henan Tobacco Industry (Hongqi Canal^®^ Filter tip cigarette; tobacco type, tar: 10 mg; nicotine content: 1.0 mg; carbon monoxide: 12 mg, Zhengzhou, China). *Klebsiella pneumoniae* (strain ID: 46114) was obtained from the National Center for Medical Culture Collection (CMCC, Beijing, China). Aminophylline was purchased from Shandong Xinhua Pharmaceutical Co., LTD. (Shandong, China). KW6002 was obtained from MedChem Express (Shanghai, China). Anti-CD4, IL-17A, CD25, and Foxp3 antibodies were obtained from eBioscience, Inc. (Affymetrix, CA, USA). Rat IL-1β, tumor necrosis factor (TNF)-α, IL-6, IL-17A, and IL-10, ELISA kits were purchased from Boster Biological Engineering (Wuhan, China). TGF-β, IL-6, and anti-CD3/CD28 antibodies were obtained from BD Biosciences (Franklin Lakes, USA). The mouse CD4^+^ CD62L^+^ T Cell Isolation Kit was obtained from Miltenyi Biotec Inc. (Miltenyi, CA, USA). Anti-A2aR, Foxp3, RORγ, p-STAT3 (Tyr705), p-STAT5 (Tyr694), STAT3, and STAT5 antibodies were purchased from Santa Cruz Biotechnology (Santa Cruz, CA, USA). HiScript QRTSuperMix and AceQ quantitative polymerase chain reaction (qPCR) SYBR Green Master Mix were purchased from Vazyme Biotech (Nanjing, China).

### Animals

Sixty-two Sprague–Dawley rats (200 ± 20 g) and 20 C57BL/6 mice were obtained from SPF biotechnology co., LTD. (Beijing, China). Animals were maintained in specific pathogen-free facilities and housed in filter-top cages with free access to food and water under a 12-h light: 12-h dark cycle in plastic cages at 25°C ± 2°C with a relative humidity of 50% ± 10%. The animal experiments were approved by the Experimental Animal Care and Ethics Committee of the First Affiliated Hospital, Henan University of Chinese Medicine.

### BYF Preparation

**Figure 1:**
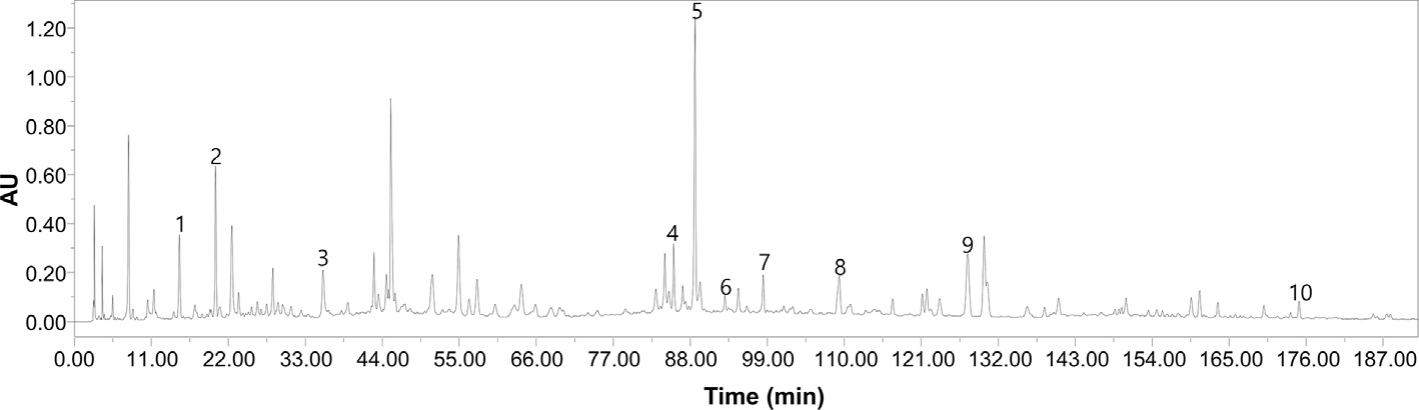
The high-performance liquid chromatography (HPLC)–UV chromatogram of Bufei Yishen Formula (BYF). Peak number and identity, 1: ursolic acid (PubChem CID: 64945); 2: bergenin (PubChem CID: 66065); 3: paeoniflorin (PubChem CID: 442534); 4: Epimedin C (PubChem CID:5748394); 5: icariin (PubChem CID:5318997); 6: Kaempferol (PubChem CID: 5280863); 7: deoxyschizandrin (PubChem CID:155256); 8: ginsenoside Rd (PubChem CID: 24721561); 9: Schisandrin (PubChem CID:23915); 10: Schisandrin B (PubChem CID:108130).

BYF is composed of 12 Chinese medicines (**Table 1**). These Chinese medicines were identified by professor Suxiang Feng and then prepared as dry extracts as described previously (Li Y. et al., 2014). High-performance liquid chromatography (HPLC) of BYF was performed, and 10 chemical constituents of BYF were identified according to the spectrograms and retention times of their standard substances using HPLC-UV (254 nm) (**Figure 1**). Finally, the per gram dry extract obtained was equivalent to 3.79 g raw medical herbs. This dry extract was given orally to COPD rats.

**Table 1:** The compositions of Bufei Yishen formula.

No.	Herbal drug	Latin scientific name	plant part(s)	Amount (g)
1	Ginseng Radix et Rhizoma	*Panax ginseng* C.A. Mey	Radix et Rhizoma	9
2	Astragali Radix	*Astragalus tibetanus* Bunge	Radix	15
3	Corni Fructus	*Cornus officinalis* Siebold & Zucc	Fructus	12
4	Lycii Fructus	*Lycium barbarum* L.	Fructus	12
5	Schisandrae Chinensis Fructus	*Schisandra arisanensis* Hayata	Fructus	9
6	Fritillariae Thunbergii Bulbus	*Fritillaria thunbergii* Miq	Bulbus	9
7	Perillae Fructus	*Perilla frutescens* (L.) Britton	Fructus	9
8	Citri Reticulatae Pericarpium	*Citrus sinensis* (L.) Osbeck	Pericarpium	9
9	Epimedii Folium	*Epimedium acuminatum* Franch	Folium	9
10	Paeoniae Rubra Radix	*Paeonia anomala* L	Radix	9
11	Pheretima	*Pheretima aspergillum* (E. Perrier)		12
12	Ardisiae Japonicae Herba	*Ardisia japonica* (Thumb.) Blume	Herba	15

The BYF dry extract was dissolved in double-distilled water at a 0.511 g/ml concentration. Then, the 70% ethanol extract was loaded on a DM101 macroporous resin column, and the 95% aqueous ethanol elution fraction was collected. Following the collection, the extracts were concentrated to total dryness by vacuum freeze-drying, and 1.6984 g of powders were acquired. Finally, the per-gram dry extract obtained was equivalent to 30 g raw medical herbs. This dry extract was prepared for cellular experiments.

### Naïve CD4^+^ T Cell Isolation and Differentiation

Naïve CD4+ T cells from mouse spleens were isolated with magnetic beads (CD4+CD62L+ T Cell Isolation Kit II, Miltenyi Biotech). TGFβ is essential for Th17 and Treg differentiation and activates both Foxp3 and RORγt expression. However, TGFβ can exclusively induce T cell differentiation into Tregs, in which higher levels of Foxp3 inhibit RORγt transcriptional activation. In the presence of both TGF-β and IL-6, this inhibition of RORγt was abrogated, and Th17 differentiation was initiated by activating STAT3 and other distinct transcriptional programs (Korn et al., 2009; Kimura and Kishimoto, 2010). Naïve CD4+ T cells were cultured *in vitro* with anti-CD3 and anti-CD28 (2 μg/ml) under Th17 (TGF-β, 5 ng/ml; IL-6, 20 ng/ml) or Treg (TGF-β, 5 ng/ml) differentiation conditions in the presence/absence of BYF (60 μg/ml) or KW6002 (0.1 μM). After stimulation for 3 days, the cells were harvested for analysis.

### COPD Rat Model and Drug Administration

COPD rat preparation and drug administration were described previously (Li Y. et al., 2012). Briefly, rats were maintained in a closed chamber exposed to tobacco from weeks 1–12 and repeated *K. pneumoniae* infections from weeks 1–8. The COPD rats were orally treated with normal saline, BYF (4.4 g/kg), KW6002 (2 mg/kg), BYF (4.4 g/kg) + KW6002 (2 mg/kg) or aminophylline (2.3 mg/kg) every day during weeks 9–20. Normal saline was also orally administrated to control rats. On week 20, all rats were anesthetized and sacrificed to obtain lung tissues, blood, and bronchoalveolar lavage fluid. All experimental procedures were approved by the Experimental Animal Care and Ethics Committee of the First Affiliated Hospital, Henan University of Chinese Medicine. The medium BYF dose (4.4 g/kg) was calculated using the formula D rat=D human×(I rat/I human)×(W rat/W human)2/3, where D=dose, I=body shape index, and W=body weight.

### Pulmonary Function and Histological Analyses

To assess pulmonary function, tidal volume (TV), peak expiratory flow (PEF), and maximum minute ventilation (MMV) were measured using unrestrained plethysmography (Buxco Inc., Wilmington, NC, USA) every four weeks during weeks 0–20.

The left lower lobe was fixed with 10% formalin neutral buffer solution, embedded in paraffin, cut into 4-μm sections, and stained with Mayer’s hematoxylin and 1% eosin alcohol solution (H&E staining).

### Cytokine ELISA Assays

IL-1β, TNF-α, IL-6, IL-17A, and IL-10 serum levels in the rats were measured with ELISA kits according to the manufacturer’s instructions. All determinations were performed in triplicate.

### Flow Cytometry

Cell suspensions containing 10^6^ cells/ml were prepared. Cells were stained with FITC-anti-CD4 and APC-anti-CD25 antibodies, fixed, permeabilized with 0.1% Triton, and stained with PE-anti-Foxp3 and PE-anti-IL-17A antibodies. Isotype controls were included in all experiments to adjust the background signal. T cells were analyzed using a FACS CantoTM II (BD Biosciences, San Jose, CA, USA), and the results were analyzed with FlowJo7.6.1 software (Tree Star, USA).

### Quantitative Real-Time PCR

Total RNA was extracted from spleens or cultured cells using Trizol according to the manufacturer’s instructions (Invitrogen Corp.). Subsequently, RNA was reversed transcribed into cDNA using HiScript II Reverse Transcriptase (Vazyme, Nanjing, China). Real-time quantitative PCR was performed using a real-time reverse transcription (RT)-PCR (Applied Biosystems, CA, USA) based on general fluorescence detection by SYBR Green. For mRNA transcript analysis, gene-specific values were normalized to the *Actb* gene.

### Western Blot Assay

Proteins were extracted from spleens, lung or cultured cells lysed with RIPA lysis buffer (Solarbio life sciences, Beijing, China) on ice and centrifuged, and then supernatants were collected. Total protein was mixed with SDS sample buffer, boiled, separated by SDS-PAGE, and transferred to PVDF membranes, which were blocked with non-fat milk for 2 h. PVDF membranes were incubated overnight at 4°C with specific primary antibodies. After being washed three times with buffer, the membranes were incubated with HRP-conjugated secondary antibodies for 2 h. Finally, the bands were visualized by film exposure with an ECL reagent.

### Statistical Analysis

All values were expressed as means ± standard errors of the means (S.E.M.). Statistical differences were assessed by one-way analysis of variance (ANOVA), followed by Tukey’s *post hoc* test. Values of P < 0.05 were considered to indicate significant differences.

## Results

### Effect of BYF on Th17 and Treg Cell Differentiation *In Vitro*

**Figure 2:**
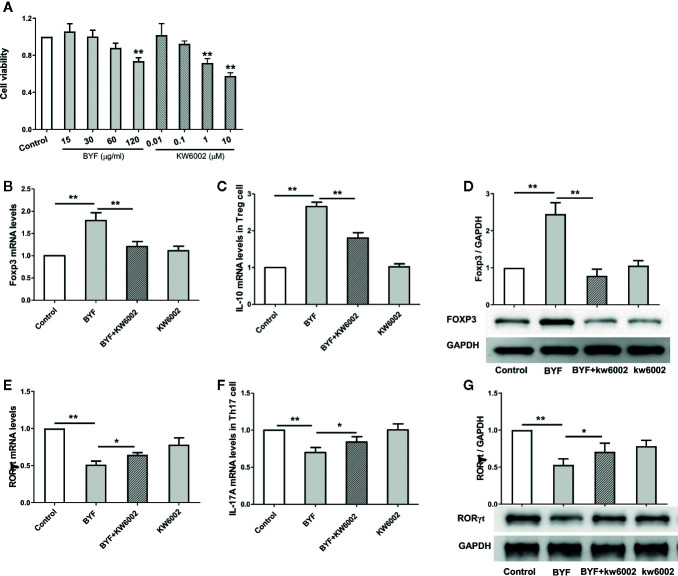
Effect of Bufei Yishen formula (BYF) on Th17 and Treg cell differentiation. Naïve CD4+ T cells were isolated from spleens of mice, and treated with Th17 differentiation conditions or Treg differentiation conditions in the presence or absence of BYF (60 μg/ml) or KW6002 (0.1 μM) for 3 days. **(A)** the effect of BYF and KW6002 on cell viability of Naïve CD4+ T cells. **(B)** The mRNA levels for Foxp3. **(C)** The mRNA levels IL-10. **(D)** The protein level of Foxp3. **(E)** RORγt mRNA; **(F)** IL-17 mRNA; **(G)** RORγt protein. *P < 0.05, **P < 0.01.

To explore how BYF restores the Th17/Treg balance, we first investigated the biological effect of BYF on Th17 cell differentiation. Naïve CD4^+^ T cells were exposed to anti-CD3, anti-CD28, TGF-β, and IL-6 to promote their differentiation into Th17 cells in the presence of BYF. IL-6 and TGF-β cotreatment activates RORγt, which is the critical transcription factor driving Th17 differentiation (Kimura and Kishimoto, 2010). BYF obviously suppressed RORγt and IL-17A mRNA expression and RORγt protein levels, suggesting that BYF inhibited Th17 cell differentiation (**Figure 2**). To investigate whether A2aR contributes to the BYF-mediated inhibition of Th17 cell differentiation, naïve CD4+ T cells were exposed to Th17-stimulatory factors and KW6002 (A2aR antagonist) in the presence of BYF. The results showed that KW6002 significantly suppressed the inhibitory effect of BYF on RORγt and IL-17 expression.

Treg cells exhibit immunosuppressive eﬀects by expressing anti-inﬂammatory cytokines, such as IL-10. Additionally, TGFβ can selectively induce T cell differentiation into Tregs, which express higher Foxp3 levels (Kimura and Kishimoto, 2010). To further explore the effect of BYF on Treg cell differentiation, naïve CD4+ T cells were induced with anti-CD3, anti-CD28, and TGF-β in the presence of BYF. BYF markedly increased the expression of the Treg markers Foxp3 and IL-10. Additionally, KW6002 obviously reduced the BYF-mediated increase in IL-10 and Foxp3 levels. These data demonstrated that BYF restored the Th17/Treg balance by regulating Th17 and Treg differentiation, in which A2aR might play an important role.

### Effect of BYF on A2aR Expression in Th17 and Treg Cells

**Figure 3:**
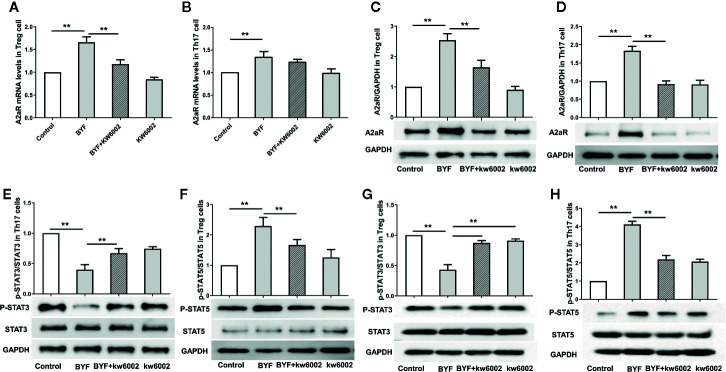
Effect of Bufei Yishen formula (BYF) on A2aR and STAT3/5 in Th17 and Treg cells. Naïve CD4+ T cells were treated with Th17 differentiation conditions or Treg differentiation conditions in the presence or absence of BYF (60 μg/ml) or KW6002 (0.1 μM) for 3 days. The mRNA levels of A2aR in Treg **(A)** and Th17 cells **(B)**; the protein levels of A2aR in Treg **(C)** and Th17 cells **(D)**; the protein levels of STAT3 and p-STAT3 in Th17 cells **(E)** and Treg cells **(G)** and STAT5 and p-STAT5 in Treg cells **(F)** and Th17 cells **(H)**. **P < 0.01.

Next, we investigated the effect of BYF on A2aR expression during Th17 and Treg differentiation. BYF significantly increased A2aR RNA and protein levels in Th17 and Treg cells, which were suppressed by KW6002 (**Figure 3**). These data suggested that BYF increased A2aR expression *via* a positive feedback mechanism.

Previously, we showed that BYF regulated STAT3 and STAT5 phosphorylation. STAT3 activation regulates IL-6-mediated RORγt expression and IL-17 production. Activated STAT5 binds to the Foxp3 gene promoter to regulate Treg cell activity (Korn et al., 2009; Kimura and Kishimoto, 2010). Here, we found that BYF decreased the STAT3 phosphorylation level and increased STAT5 phosphorylation in Th17 and Treg cells, which were inhibited by KW6002 (**Figures 3E–H**).

### Effect of A2aR Antagonist on the BYF-Reduced Severity of COPD

**Figure 4:**
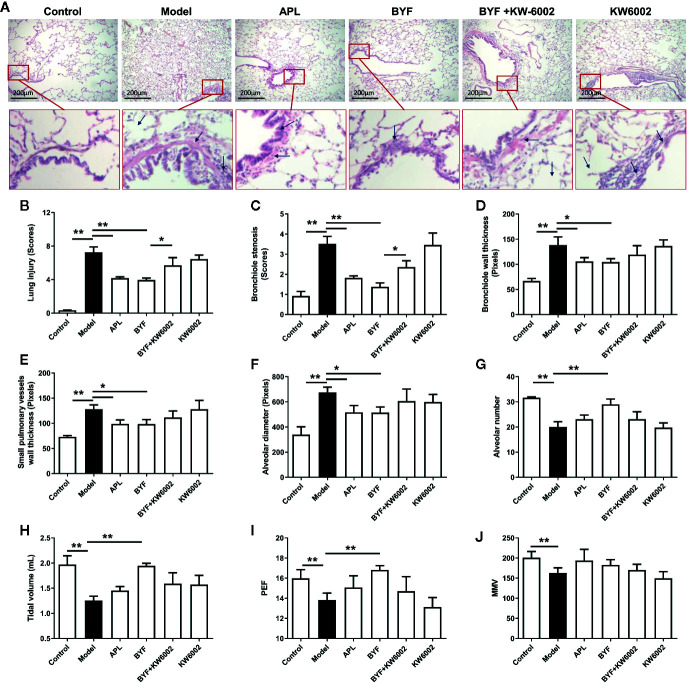
Effect of cotreatment of Bufei Yishen formula (BYF) and A2aR antagonist on chronic obstructive pulmonary disease (COPD) in rats. COPD rats were orally administrated with BYF, KW6002 and aminophylline. **(A)** Histopathological changes of lung tissues of each group (HE staining, magniﬁcation, ×100). **(B)** Statistics of lung injury scores, **(C)** bronchiole stenosis, **(D)** bronchial wall thickness, **(E)** Small pulmonary vessels wall thickness, **(F)** alveolar diameter, **(G)** alveolar number, **(H)** Tidal volume (TV), **(I)** Peak expiratory flow (PEF), **(J)** Maximum minute ventilation (MMV). All data are presented as mean ± SEM. N = 6 for each group. *P < 0.05, **P < 0.01.

**Figure 5:**
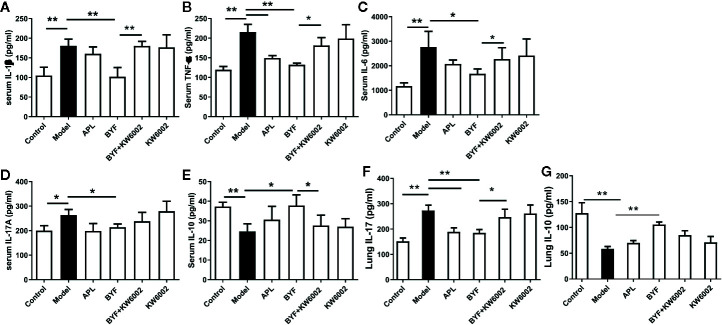
Effect of cotreatment of Bufei Yishen formula (BYF) and A2aR antagonist on cytokine profiles in chronic obstructive pulmonary disease (COPD) rats. COPD rats were orally administrated with BYF, KW6002 and aminophylline. Cytokine levels of f IL-1β, TNF-α, IL-6, IL-17A, and IL-10 in serum or lung tissues were detected by ELISA. The results were independently replicated. The values are presented as the means ± SEM (n= 6 mice per group). *P < 0.05, **P < 0.01.

KW6002 and BYF were administered to the COPD rats to verify the relevance of A2aR activation and amelioration of COPD by BYF. The results showed that BYF significantly reduced the severity of COPD. However, KW6002 diminished the therapeutic effect of BYF on COPD, as indicated by the lung injury scores, bronchiole wall thickness, small pulmonary vessels wall thickness, bronchiole stenosis, alveolar diameters, increase in alveolar number, and lung functions (TV, PEF, and MMV) (**Figures 4A–J**). Additionally, KW6002 significantly reduced the inhibitory effect of BYF on IL-1β, TNF-α, and IL-6 levels (proinflammatory cytokines) and Th17-related IL-17, as well as the increase in Treg-related IL-10 (**Figures 5A–G**). These results suggested that A2aR activation was critical for the anti-COPD effect of BYF.

### Effect of KW6002 on the BYF-Restored Th17/Treg Balance in COPD Rats

**Figure 6:**
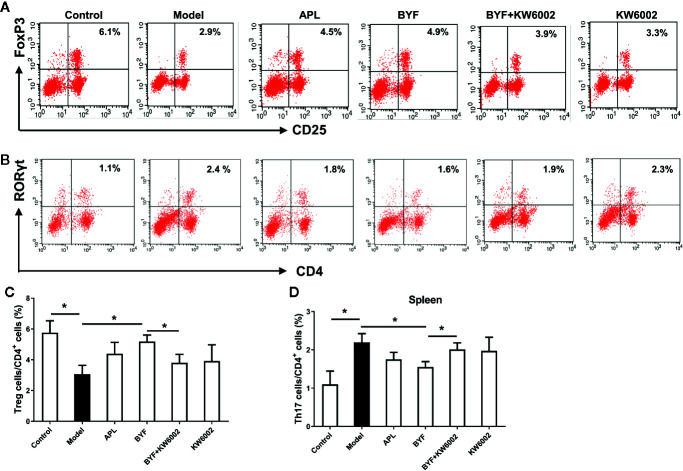
Effect of cotreatment of Bufei Yishen formula (BYF) and A2aR antagonist on the balance of Th17/Treg cells in spleens of chronic obstructive pulmonary disease (COPD). COPD rats were orally administrated with BYF, KW6002 and aminophylline. The proportion of Treg **(A)** and Th17 cells **(B)** was detected. Statistics of the proportion of Treg **(C)** and Th17 cells **(D)** were analyzed. The values are presented as the means ± SEM (n=6 mice per group). *P < 0.05.

**Figure 7:**
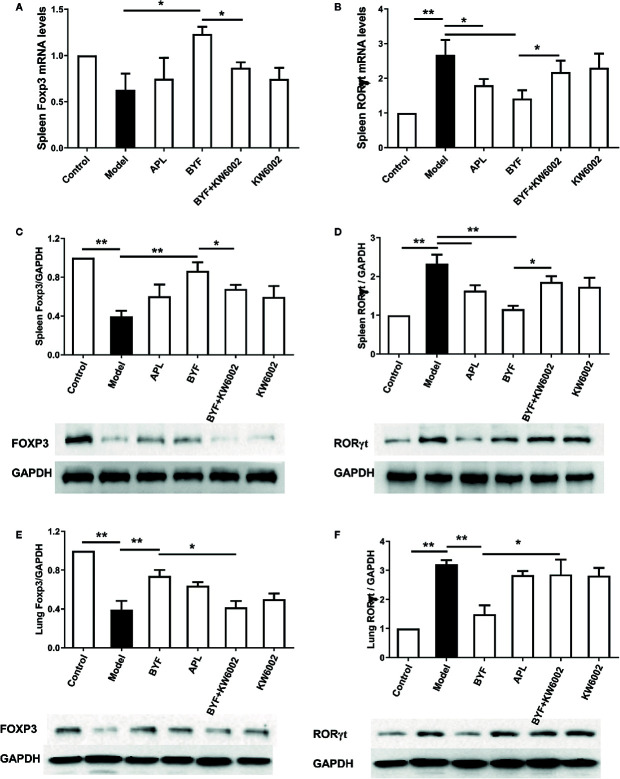
Effect of cotreatment of Bufei Yishen formula (BYF) and A2aR antagonist on the mRNA levels of RORγt and Foxp3 in the spleens of chronic obstructive pulmonary disease (COPD) rats. The mRNA levels for Foxp3 **(A)** and RORγt **(B)** in the spleens were analyzed by a quantitative polymerase chain reaction (PCR) assay. The protein levels for Foxp3 **(C)** and RORγt **(D)** in the spleens, and Foxp3 **(E)** and RORγt **(F)** in the lung were detected by Western blotting. The values are presented as the means ± SEM (n=6 mice per group). *P < 0.05, **P < 0.01.

**Figure 8:**
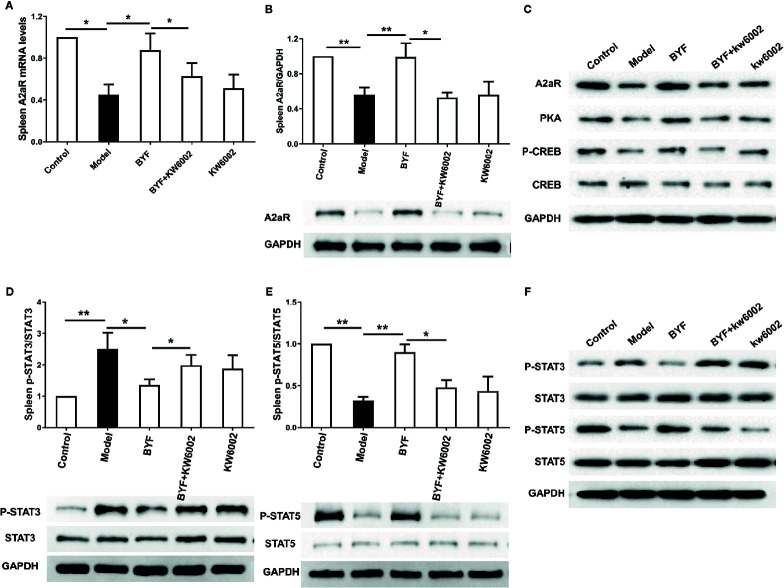
Effect of cotreatment of Bufei Yishen formula (BYF) and A2aR antagonist on expression of A2aR and the phosphorylation of STAT3 and STAT5 in the spleens of chronic obstructive pulmonary disease (COPD) rats. The mRNA level of A2aR in spleen was detected **(A)**. Expression of A2aR in spleen **(B)** and A2aR signal proteins including A2aR, protein kinase A (PKA), p-CREB, and CRE binding (CREB) in lung proteins **(C)** were analyzed by western blotting; p-STAT3/STAT3 **(D)**, p-STAT5/STAT5 **(E)** in spleen and in lung **(F)** were analyzed by western blotting. Data was showed as the means ± SEM (n=6 mice per group). *P < 0.05, **P < 0.01.

To verify the BYF-mediated regulation of Th17 and Treg cells *via* A2aR in COPD rats, we further explored the effect of KW6002 on the BYF-restored Th17/Treg balance in rat spleens. BYF treatment obviously decreased the percentage of CD4+IL-17+ T cells and increased the percentage of CD4+CD25+Foxp3+ T cells (**Figure 6**). KW6002 reversed the decreased Th17 cell proportion and increased Treg cell proportion induced by BYF. KW6002 also reduced the inhibitory effect of BYF on RORγt expression and its promotion of Foxp3 mRNA and protein expression (**Figure 7**). Additionally, BYF increased the A2aR protein and mRNA levels in spleen tissues, and upregulated A2aR, PKA and p-CREB proteins in lung tissues, which were inhibited by cotreatment with KW6002 (**Figures 8A–C**).

To further investigate whether A2aR signals contributes to the BYF-mediated differentiation of Th17 and Treg cells, we determined the effect of KW6002 on BYF-regulated STAT3 and STAT5 phosphorylation, the essential transcription factors for Th17 and Treg differentiation, in spleen tissues. We found that BYF treatment significantly reduced STAT3 phosphorylation and enhanced STAT5 phosphorylation, which were reversed by KW6002 cotreatment (**Figures 8D–F**). These results suggested that BYF regulated the Th17/Treg cell balance by activating A2aR in COPD rats.

## Discussion

We previously showed that BYF treatment ameliorated lung function and pathological changes in COPD rats (Li et al., 2015; Li Y. et al., 2016), which was mainly attributed to the inhibition of inflammatory responses and regulation of the Th17/Treg cell balance (Zhao et al., 2018). However, the mechanisms by which BYF mediates its anti-COPD effects remained unknown. In this study, we investigated how BYF regulates the Th17/Treg cell balance and COPD in rats. The data suggested that BYF inhibited Th17 differentiation and enhanced Treg differentiation by activating A2aR. Furthermore, A2aR antagonism suppressed the changes induced by BYF in Th17 cells, Treg cells, and COPD rats.

It is well established that Th17/Treg imbalance plays an important role in COPD development and progression (Yang et al., 2011). COPD patients exhibit increased Th17 cell populations accompanied by changes in their respective cytokines. Generally, Treg cells are functionally defective in COPD patients (Li X. N. et al., 2014). In this work, we found that BYF inhibited Th17 cell differentiation and enhanced Treg cell differentiation *in vitro*, which may contribute to the anti-COPD effect of BYF.

The exact mechanism by which BYF regulates Th17 and Treg differentiation is poorly understood. Many reports suggest that A2aR stimulation increases Treg cells and decreases Th17 subtypes (Li N. et al., 2012). Here, we found that BYF increased A2aR protein levels in Th17 and Treg cells, which were suppressed by KW6002. Additionally, STAT3 activated by IL-6, IL-23, and IL-21 enhanced RORγt gene expression, the critical transcriptional factor for Th17 differentiation. STAT5 can directly bind to the Foxp3 gene to regulate the development and maintenance of Treg cells. Here, we also showed that BYF increased the reduced STAT3 phosphorylation level and increased STAT5 phosphorylation in Treg and Th17 cells, which were reversed by KW6002 cotreatment.

A2aR-knockout mice show enhanced inflammation in their bronchial airways (Mohsenin et al., 2007). A2aR agonists may provide an alternative agent for the treatment of airway inflammatory diseases (Bonneau et al., 2006; Haskó et al., 2008; Golzar and Doukky, 2014). Thus, we examined the role of A2aR in mediating the anti-COPD effect of BYF and its regulation of the Th17/Treg balance in COPD rats. The results showed that BYF could activate signaling of A2aR which leads to upregulation of PKA and CREB in lung tissues. Cotreatment with KW6002 diminished the therapeutic effect of BYF on COPD. Previously, regulation of the Th17/Treg balance was shown to play a key role in the anti-COPD effect of BYF (Zhao et al., 2018). In this work, we also demonstrated that KW6002 reversed the decrease in the Th17 cell proportion, the increase in the Treg proportion, and changes in STAT3 and STAT5 phosphorylation and A2aR signaling induced by BYF in COPD rats. These data demonstrate that activating A2aR plays a critical role in the therapeutic effect of BYF.

In conclusion, these results demonstrated that BYF exerted its anti-COPD efficacy by restoring the Th17/Treg balance *via* activating A2aR, which may help to elucidate the underlying immunomodulatory mechanism of BYF and provide evidence for its clinical application in COPD treatment.

## Data Availability Statement

The raw data supporting the conclusions of this article will be made available by the authors, without undue reservation, to any qualified researcher.

## Ethics Statement

The animal study was reviewed and approved by Experimental Animal Care and Ethics Committee of the First Affiliated Hospital, Henan University of Chinese Medicine.

## Author Contributions

JL and PZ designed the outline of the study. PZ, XL, YT, and SF performed experiments, conceived the study, draft and revised the manuscript. DZ, ZR, XL, and HD were involved performing experiments, acquisition of data and statistical analysis. All authors contributed to the article and approved the submitted version.

## Funding

The research is supported by National Natural Science Fund of China [grant number 81603473], Young Elite Scientists Sponsorship Program by CACM [grant number 2019-QNRC2-B03], the Key Program of Henan Universities [grant number 16A360004], and Project funded by China Postdoctoral Science Foundation [grant number 2016M602248].

## Conflict of Interest

The authors declare that the research was conducted in the absence of any commercial or financial relationships that could be construed as a potential conflict of interest.
