# Supplementary figures and images for: Bufei Yishen Formula Restores Th17/Treg Balance and Attenuates Chronic Obstructive Pulmonary Disease via Activation of the Adenosine 2a Receptor
Source: Front Pharmacol. 2020 Aug 7;11:1212. doi: 10.3389/fphar.2020.01212 (PMC7427463; doi:10.3389/fphar.2020.01212)

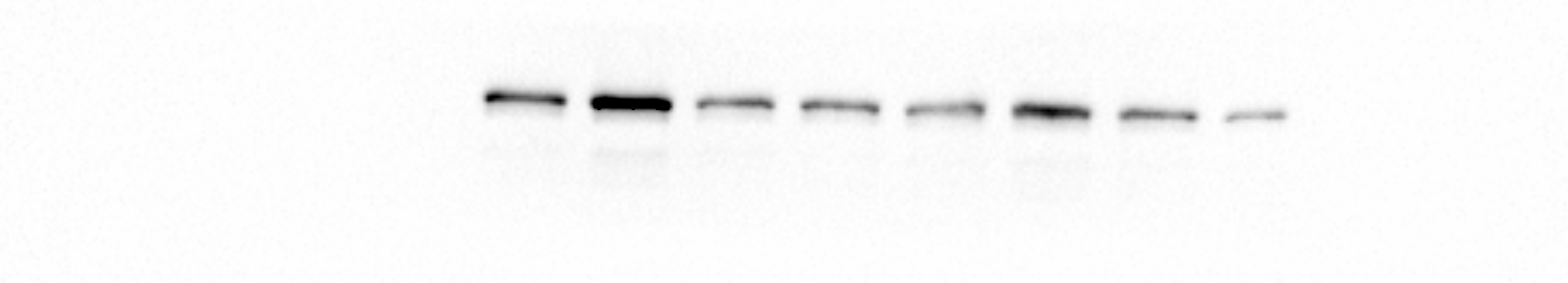

Supplement: Supplementary file 1 [file DataSheet_1.zip › Original image files/Fig 2D FOXP3.jpg]

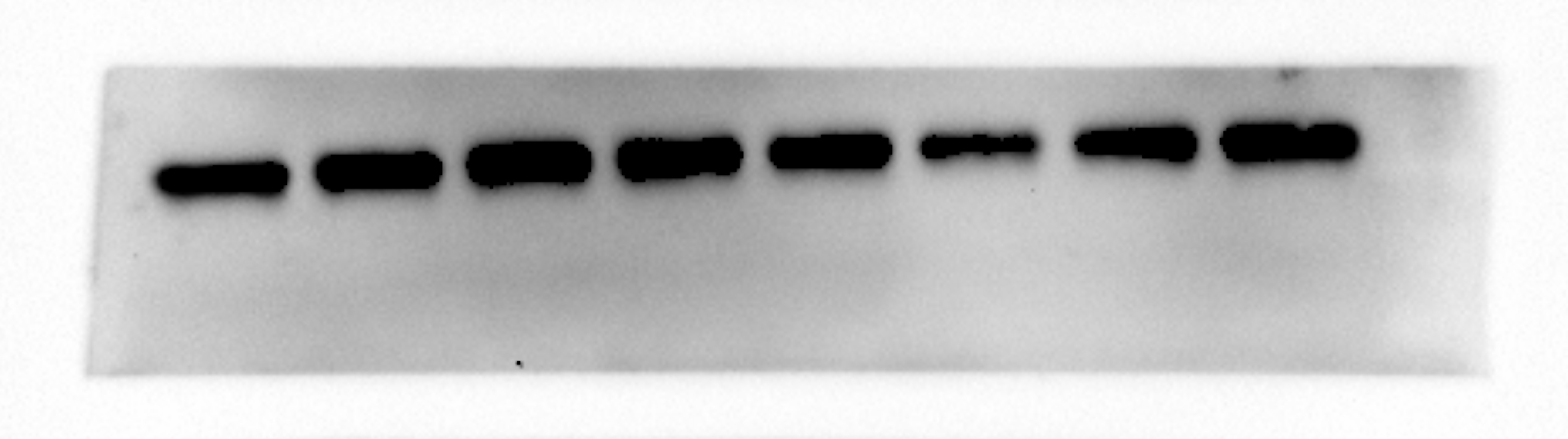

Supplement: Supplementary file 1 [file DataSheet_1.zip › Original image files/Fig 2D GAPDH.jpg]

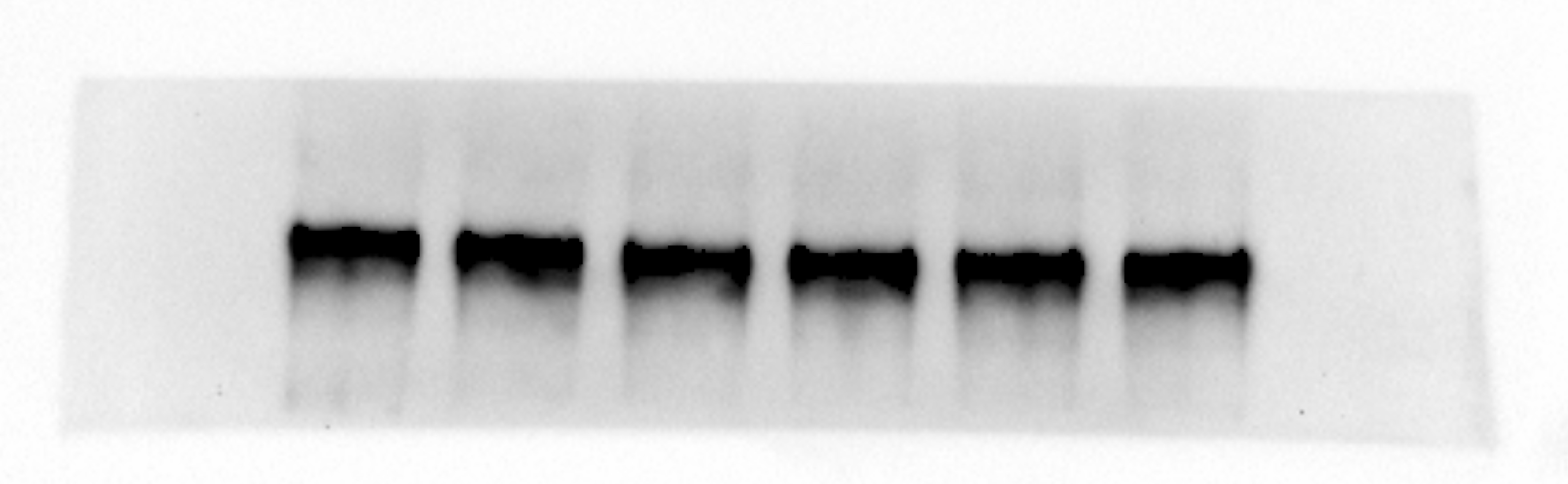

Supplement: Supplementary file 1 [file DataSheet_1.zip › Original image files/Fig 2G GAPDH.jpg]

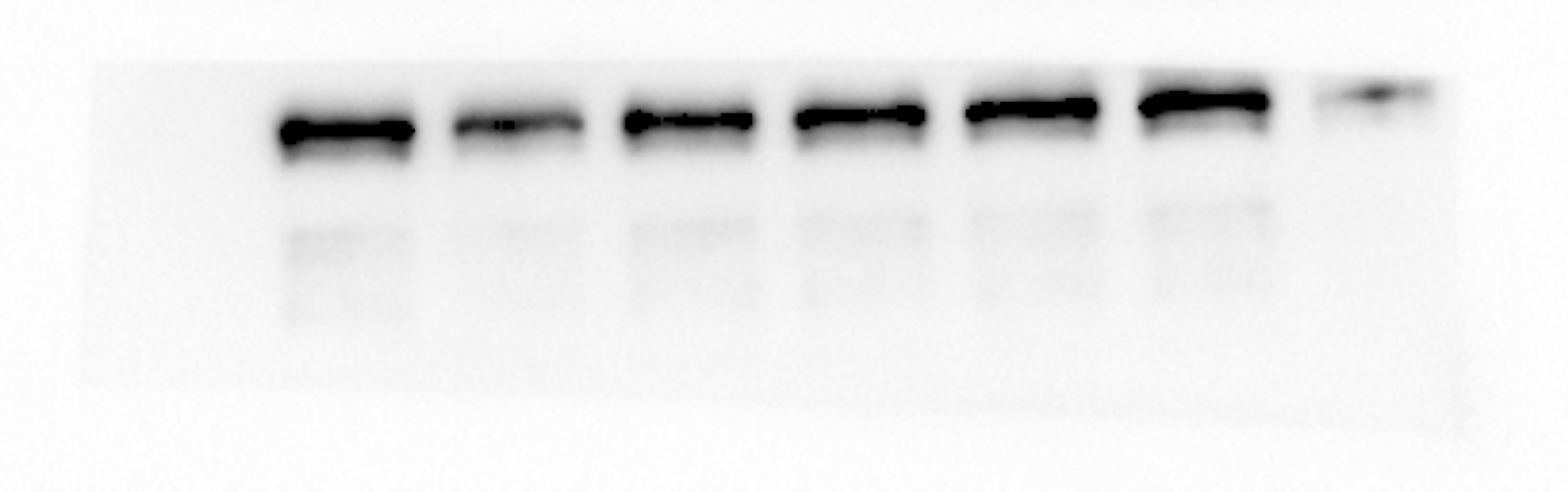

Supplement: Supplementary file 1 [file DataSheet_1.zip › Original image files/Fig 2G RORrt.jpg]

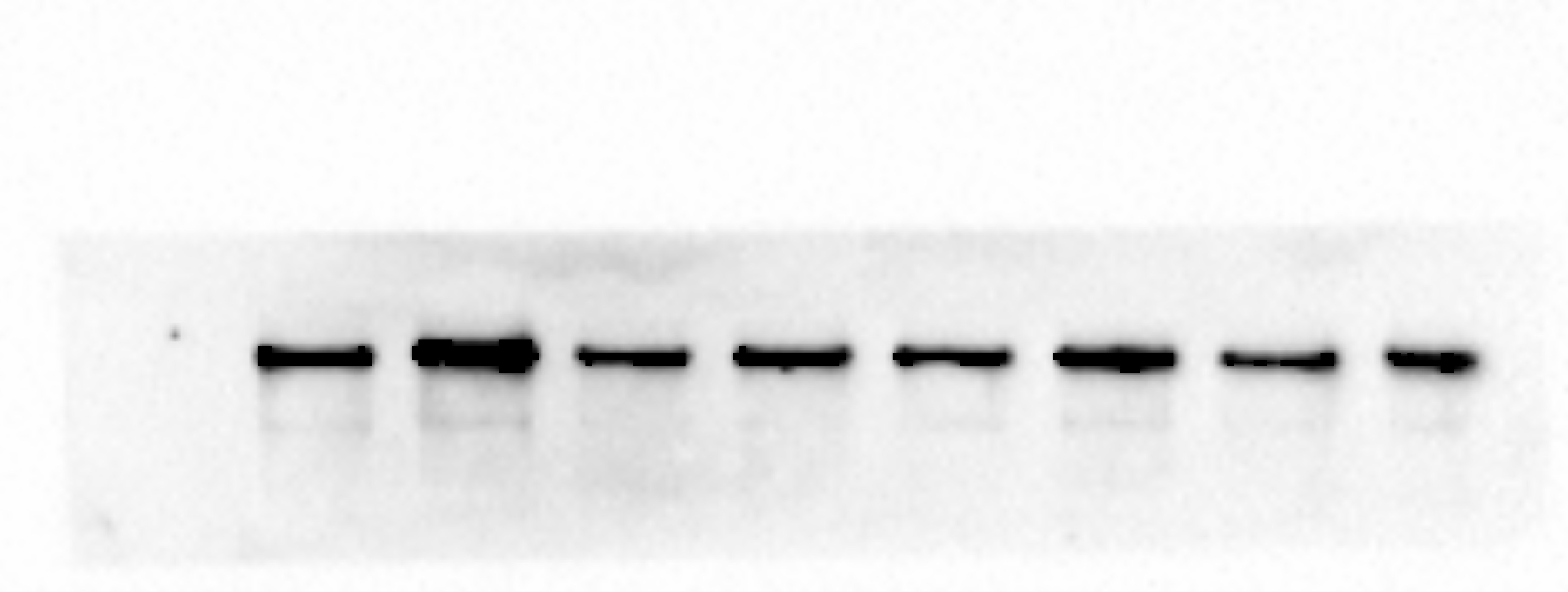

Supplement: Supplementary file 1 [file DataSheet_1.zip › Original image files/Fig 3C A2aR.jpg]

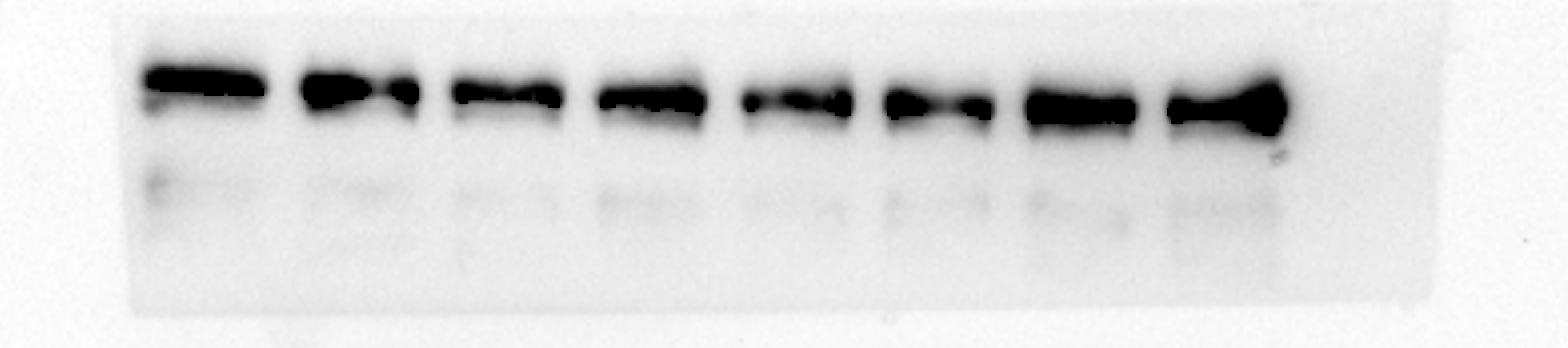

Supplement: Supplementary file 1 [file DataSheet_1.zip › Original image files/Fig 3C GAPDH.jpg]

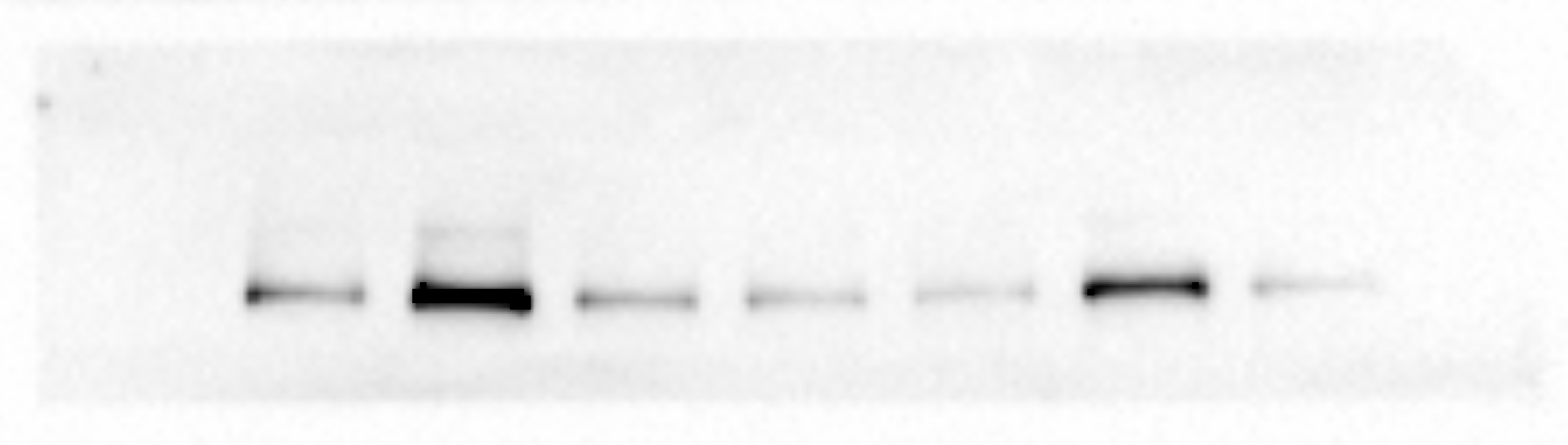

Supplement: Supplementary file 1 [file DataSheet_1.zip › Original image files/Fig 3D A2aR.jpg]

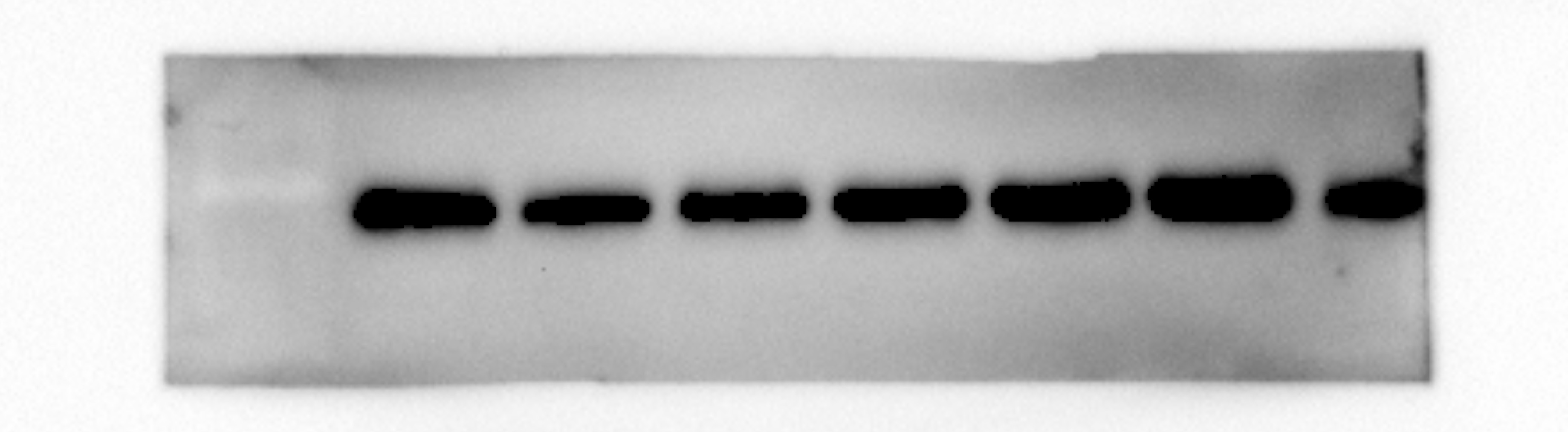

Supplement: Supplementary file 1 [file DataSheet_1.zip › Original image files/Fig 3D GAPDH.jpg]

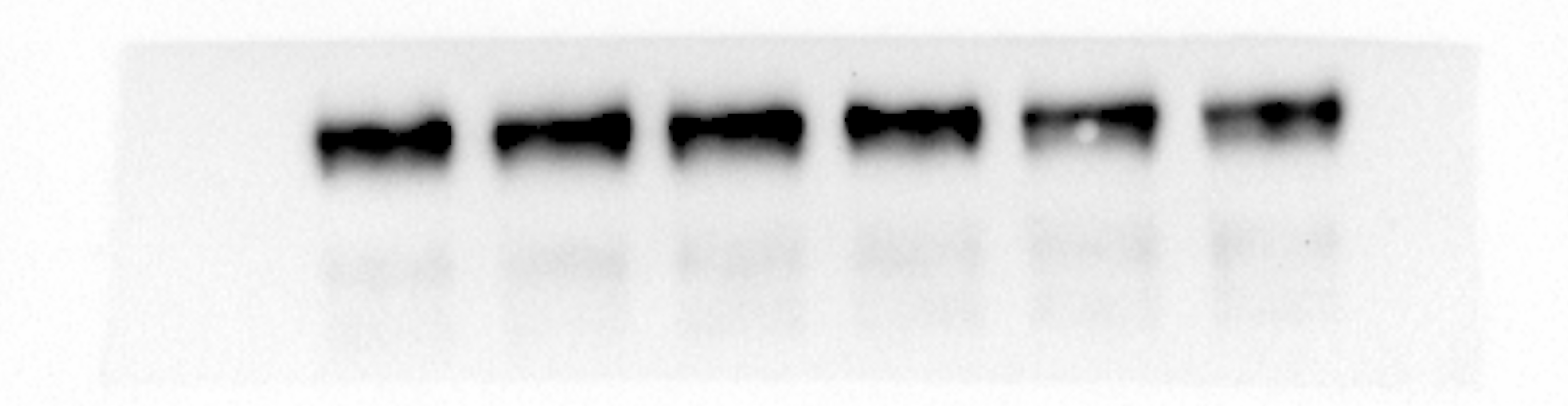

Supplement: Supplementary file 1 [file DataSheet_1.zip › Original image files/Fig 3E GAPDH.jpg]

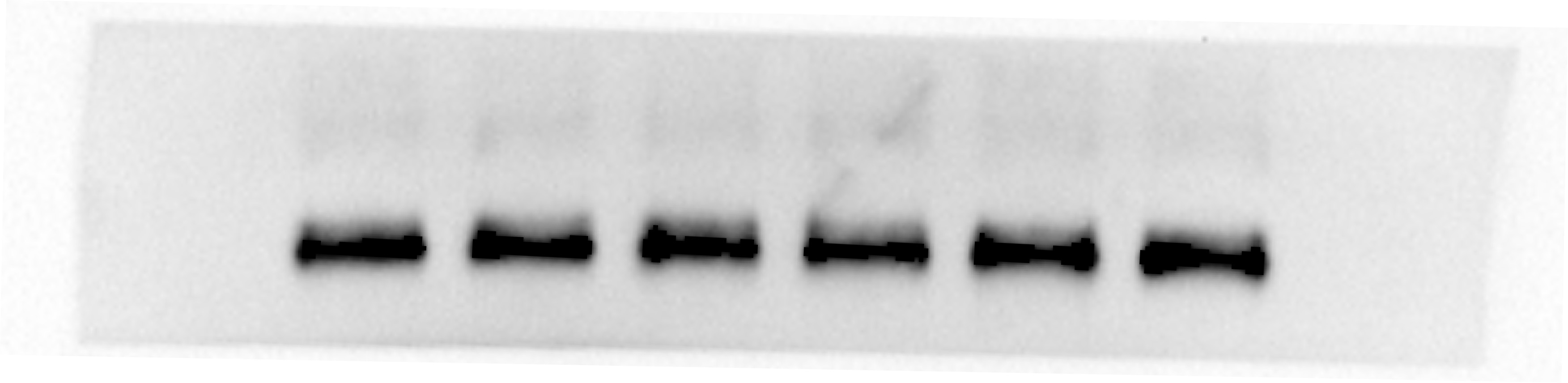

Supplement: Supplementary file 1 [file DataSheet_1.zip › Original image files/Fig 3E STAT3.jpg]

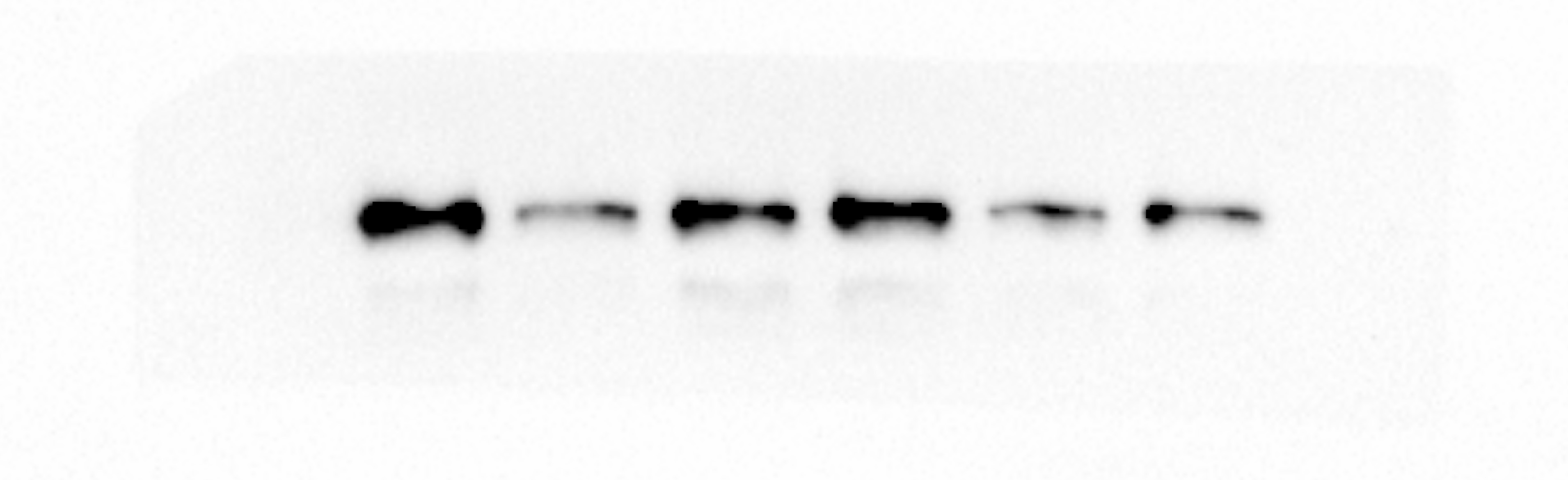

Supplement: Supplementary file 1 [file DataSheet_1.zip › Original image files/Fig 3E p-STAT3.jpg]

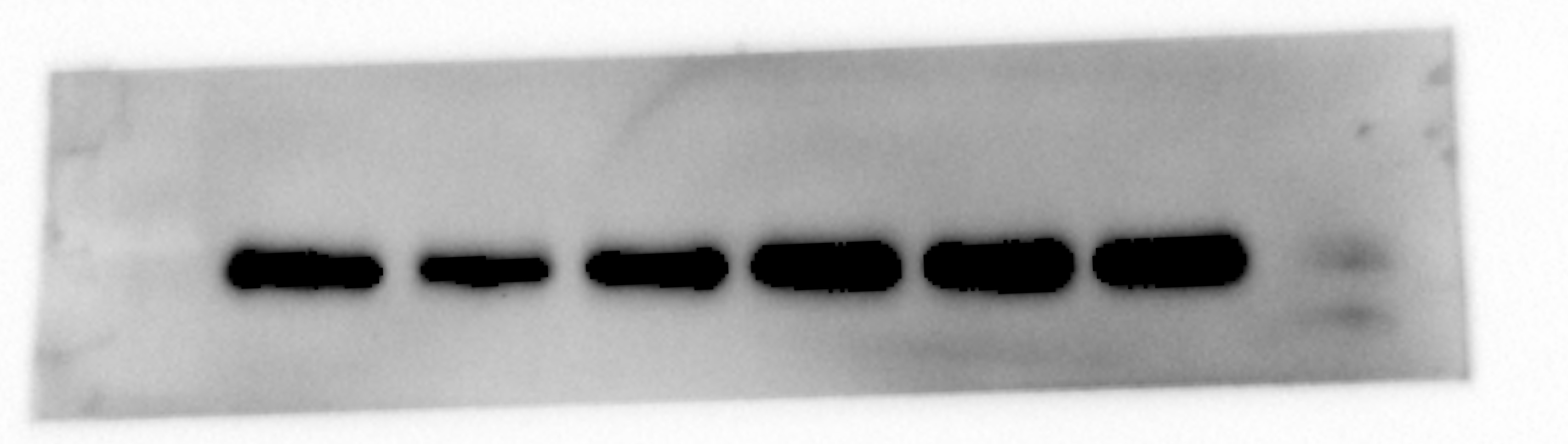

Supplement: Supplementary file 1 [file DataSheet_1.zip › Original image files/Fig 3F GAPDH.jpg]

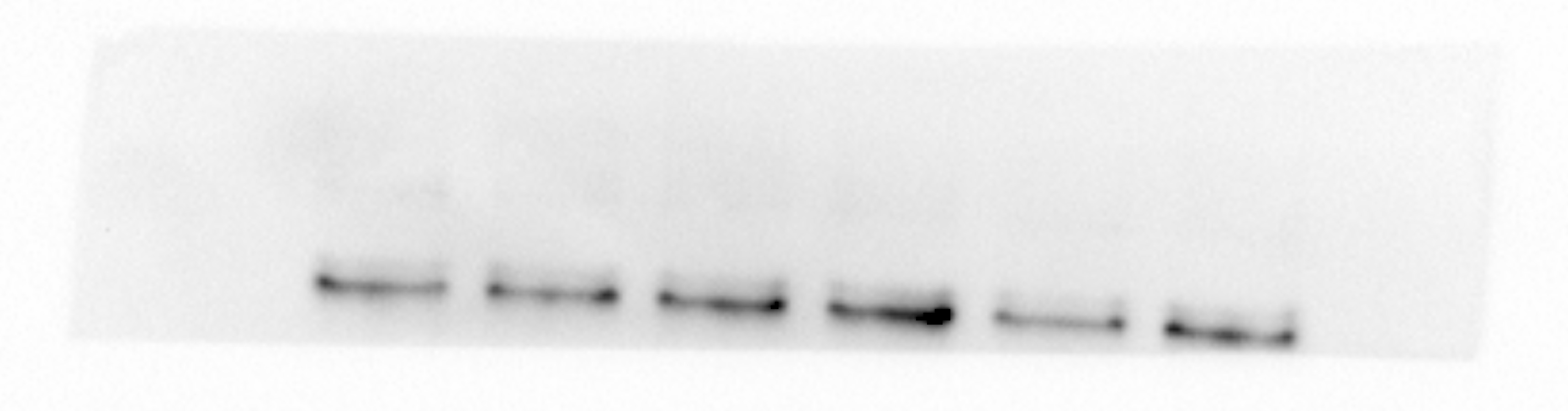

Supplement: Supplementary file 1 [file DataSheet_1.zip › Original image files/Fig 3F STAT5.jpg]

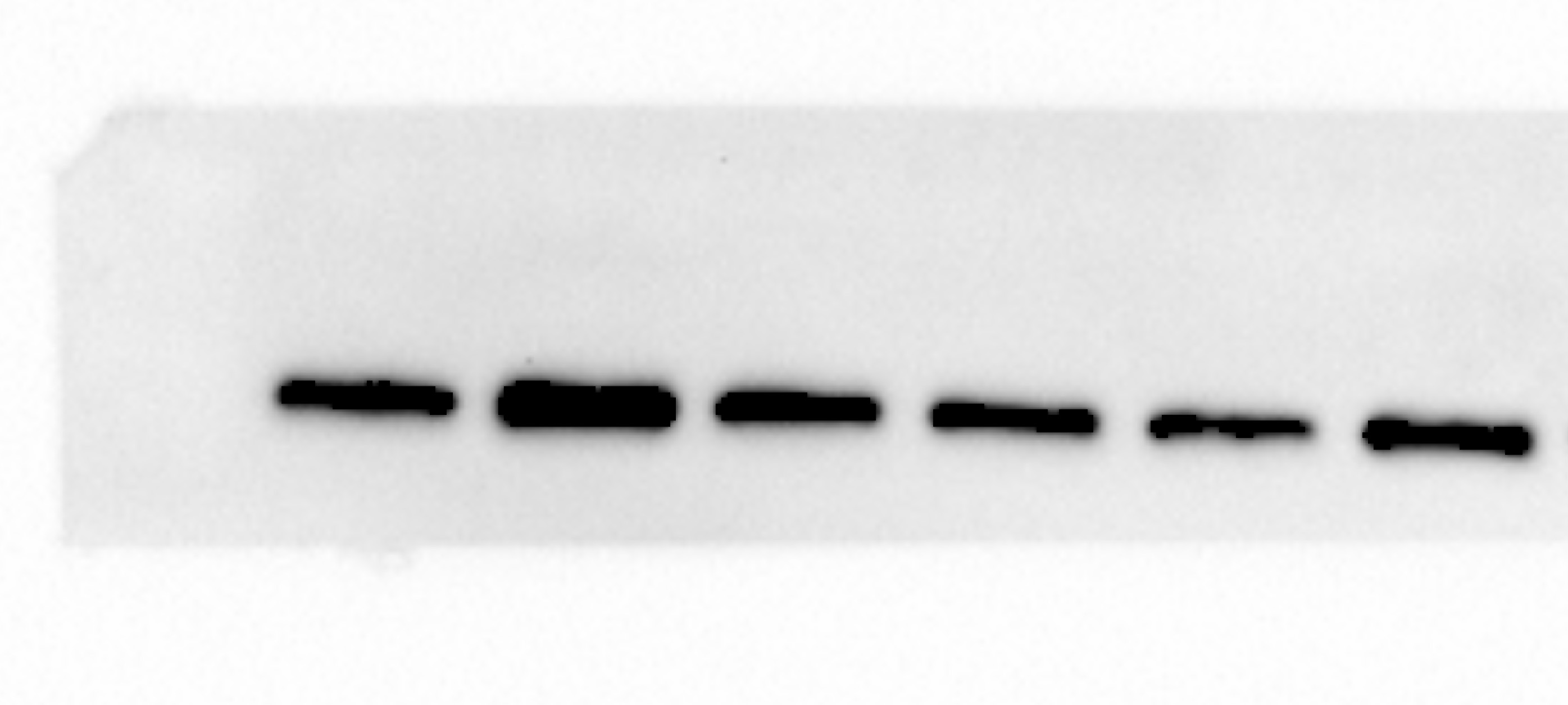

Supplement: Supplementary file 1 [file DataSheet_1.zip › Original image files/Fig 3F p-STAT5.jpg]

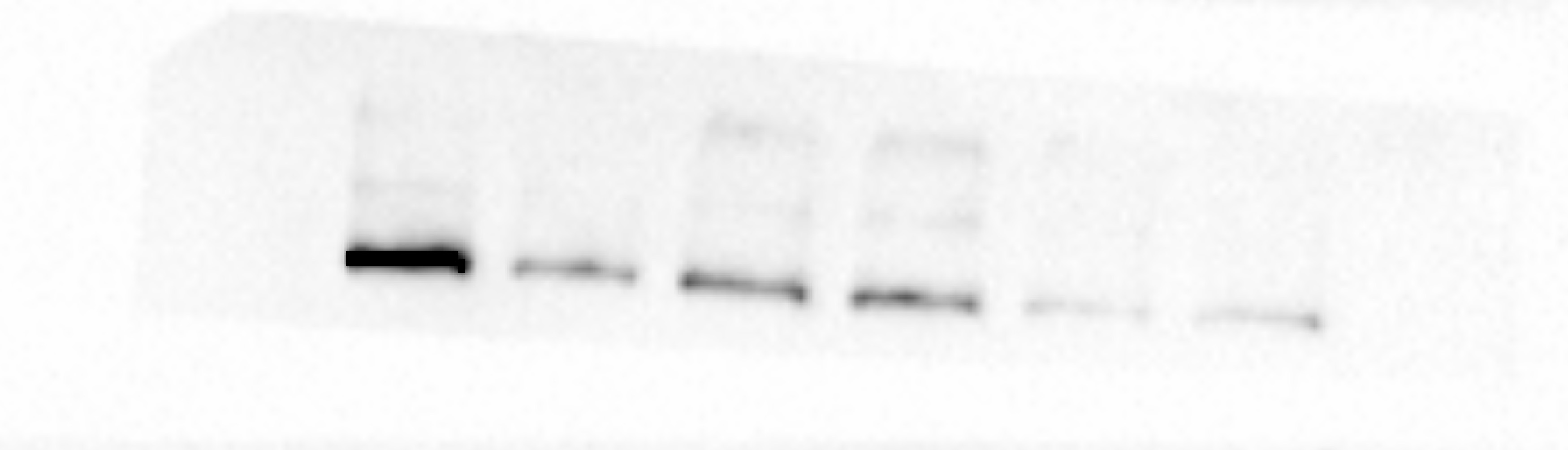

Supplement: Supplementary file 1 [file DataSheet_1.zip › Original image files/Fig 7C FOXP3.jpg]

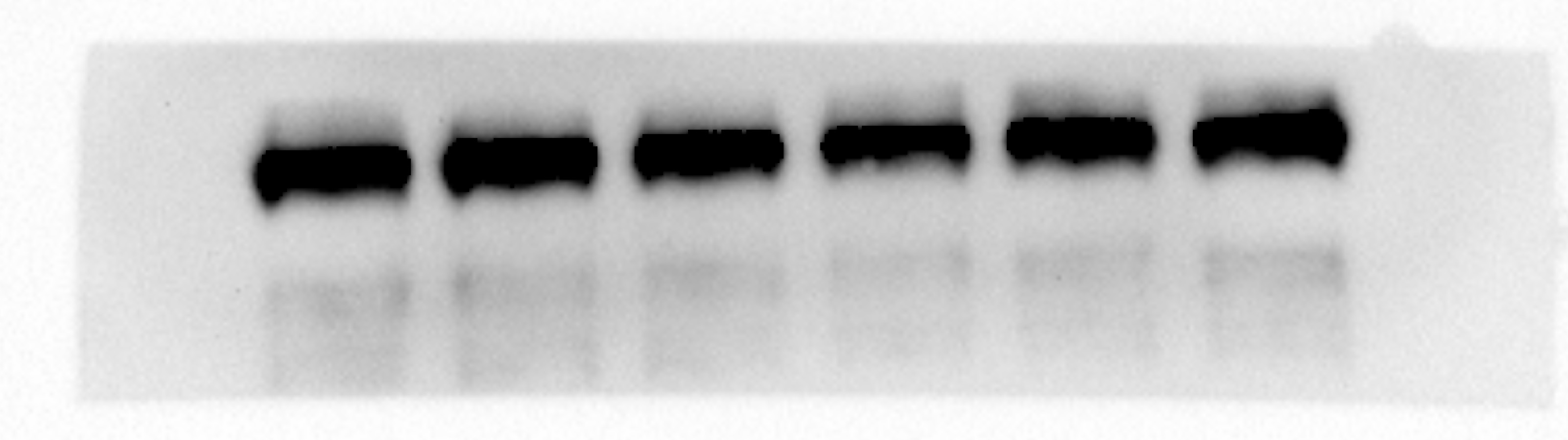

Supplement: Supplementary file 1 [file DataSheet_1.zip › Original image files/Fig 7C GAPDH.jpg]

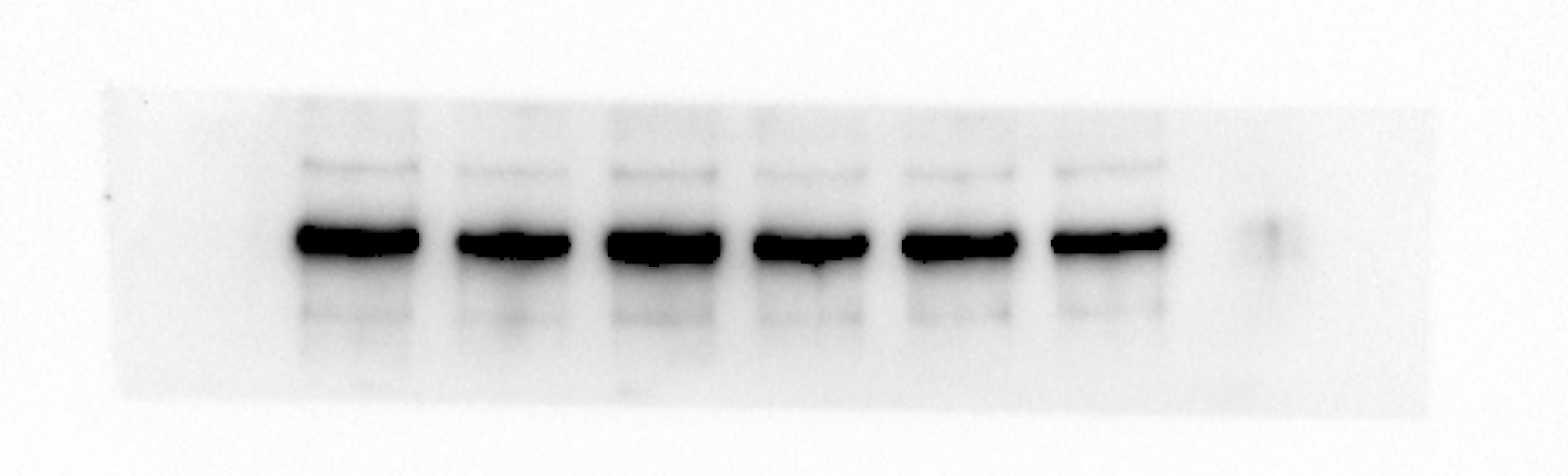

Supplement: Supplementary file 1 [file DataSheet_1.zip › Original image files/Fig 7D GAPDH.jpg]

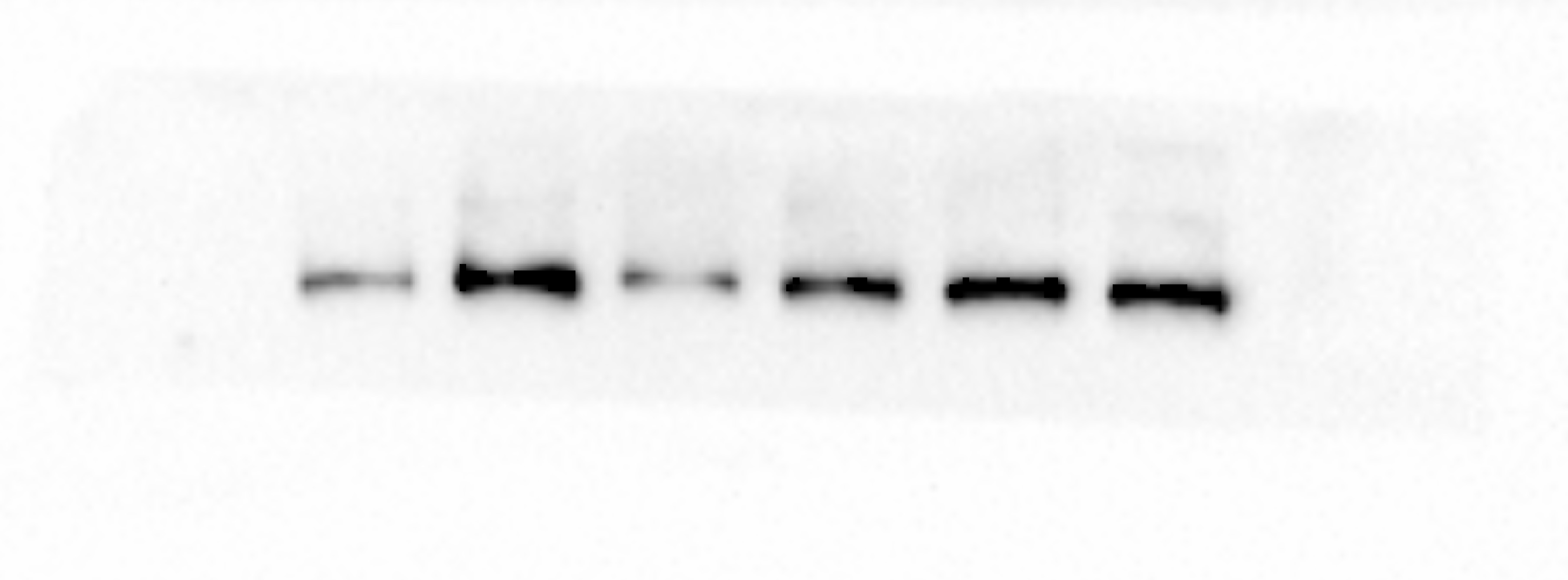

Supplement: Supplementary file 1 [file DataSheet_1.zip › Original image files/Fig 7D ROR rt.jpg]

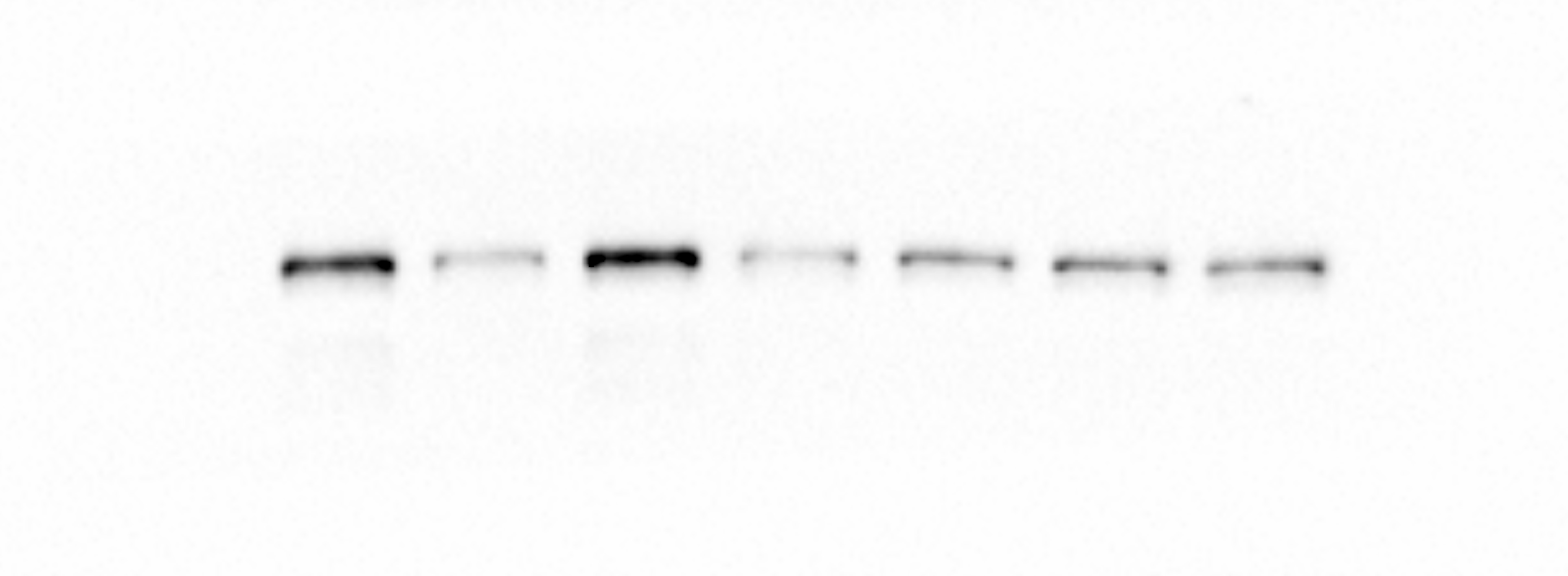

Supplement: Supplementary file 1 [file DataSheet_1.zip › Original image files/Fig 8B A2aR.jpg]

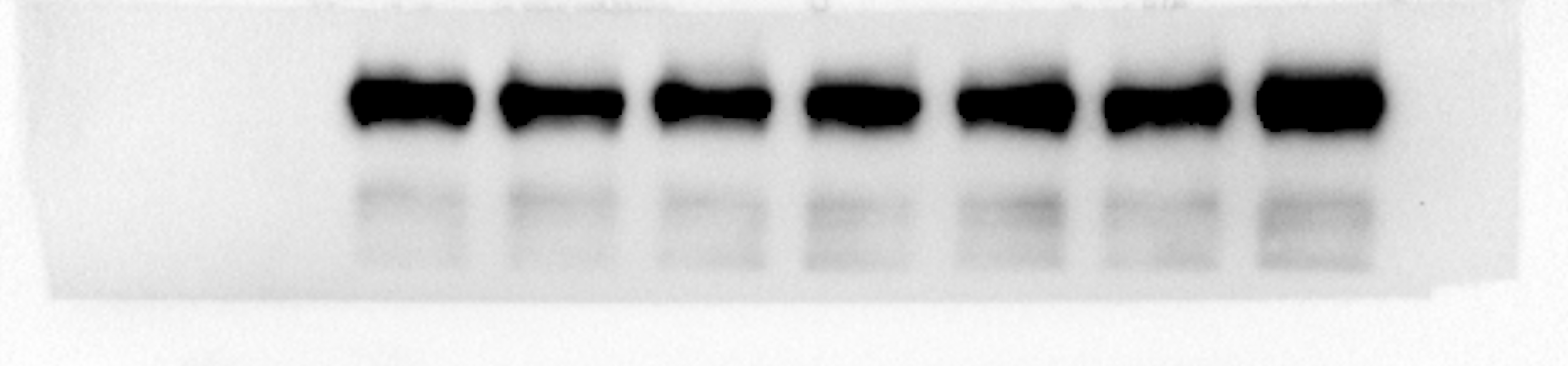

Supplement: Supplementary file 1 [file DataSheet_1.zip › Original image files/Fig 8B GAPDH.jpg]

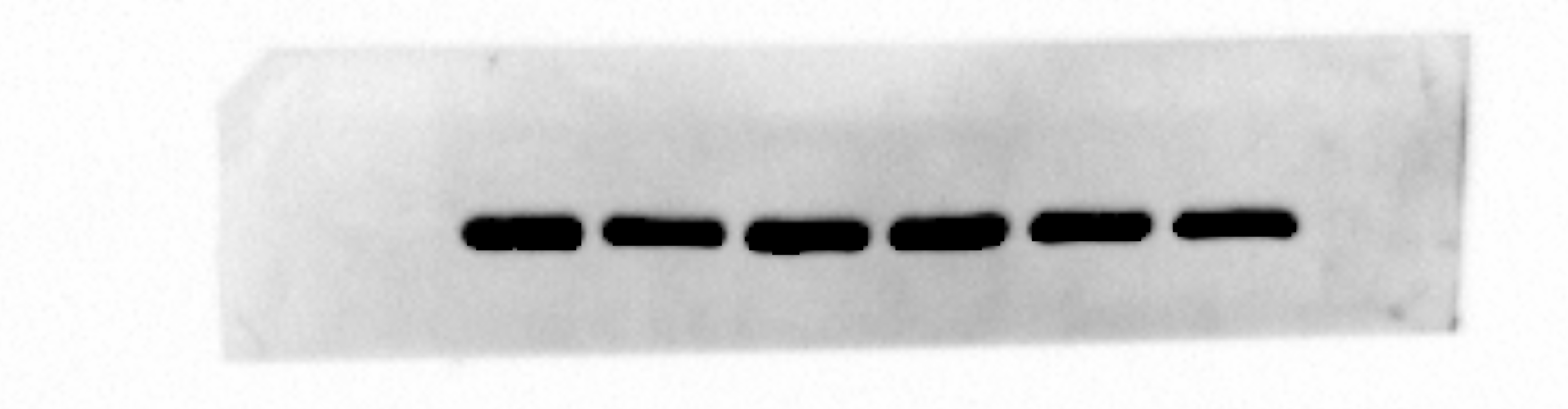

Supplement: Supplementary file 1 [file DataSheet_1.zip › Original image files/Fig 8D GAPDH.jpg]

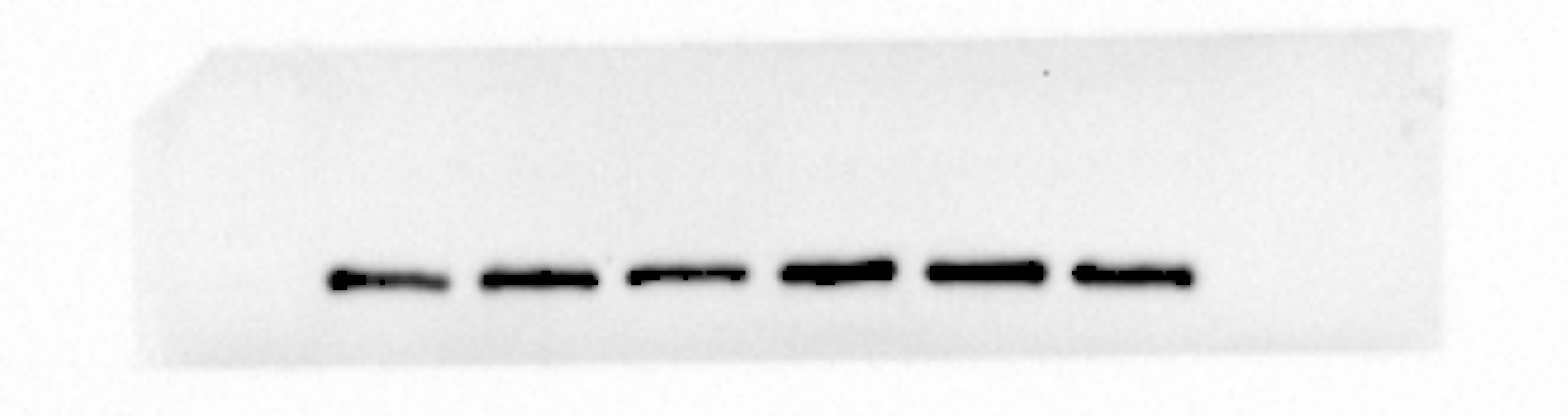

Supplement: Supplementary file 1 [file DataSheet_1.zip › Original image files/Fig 8D STAT3.jpg]

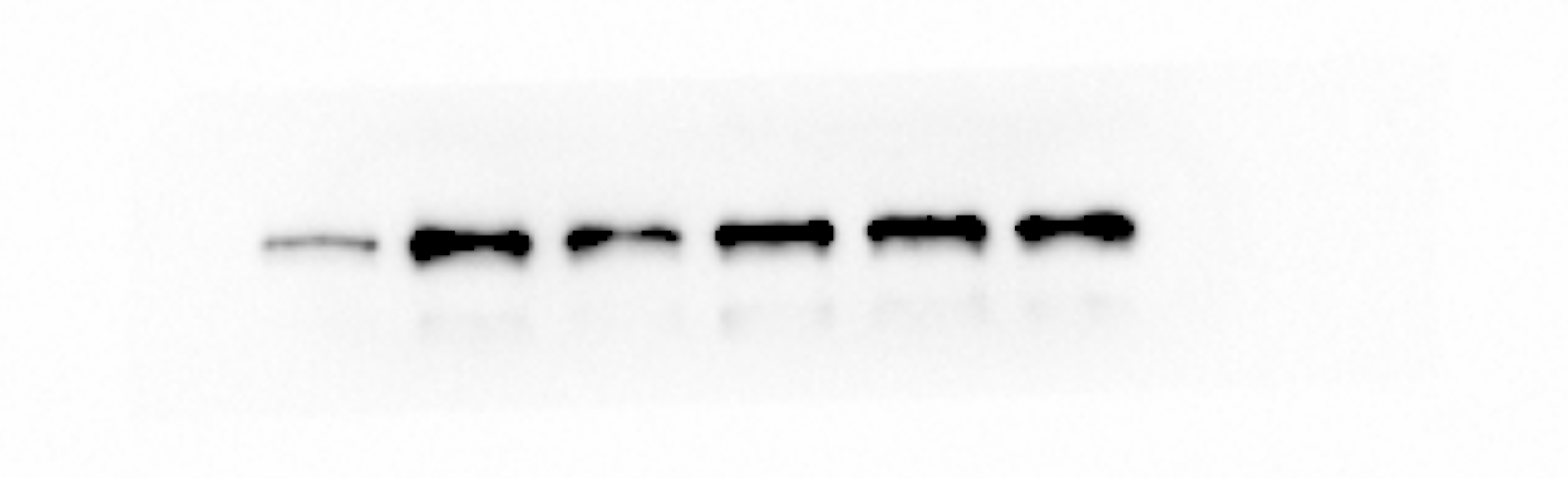

Supplement: Supplementary file 1 [file DataSheet_1.zip › Original image files/Fig 8D p-STAT3.jpg]

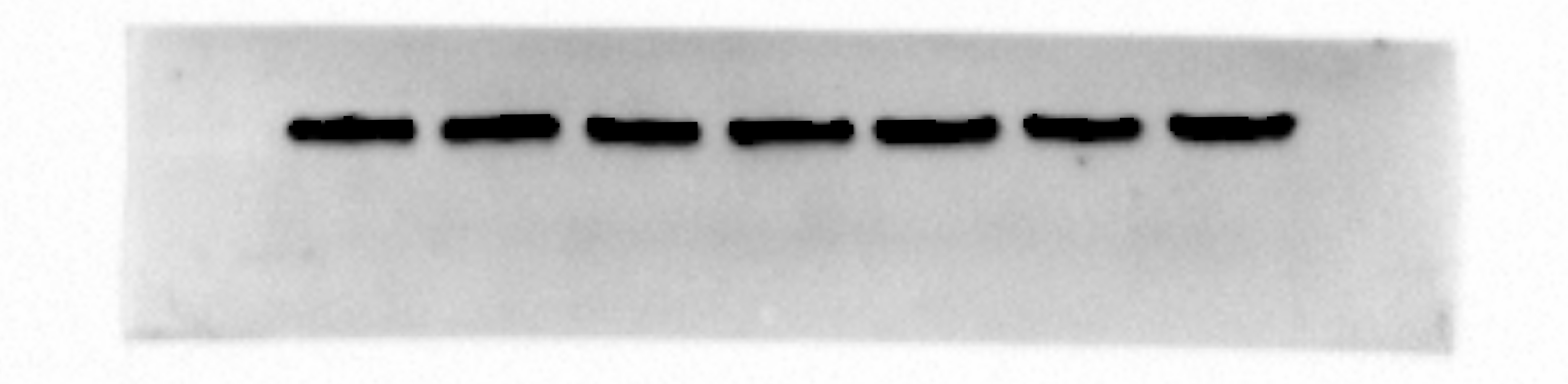

Supplement: Supplementary file 1 [file DataSheet_1.zip › Original image files/Fig 8E GAPDH.jpg]

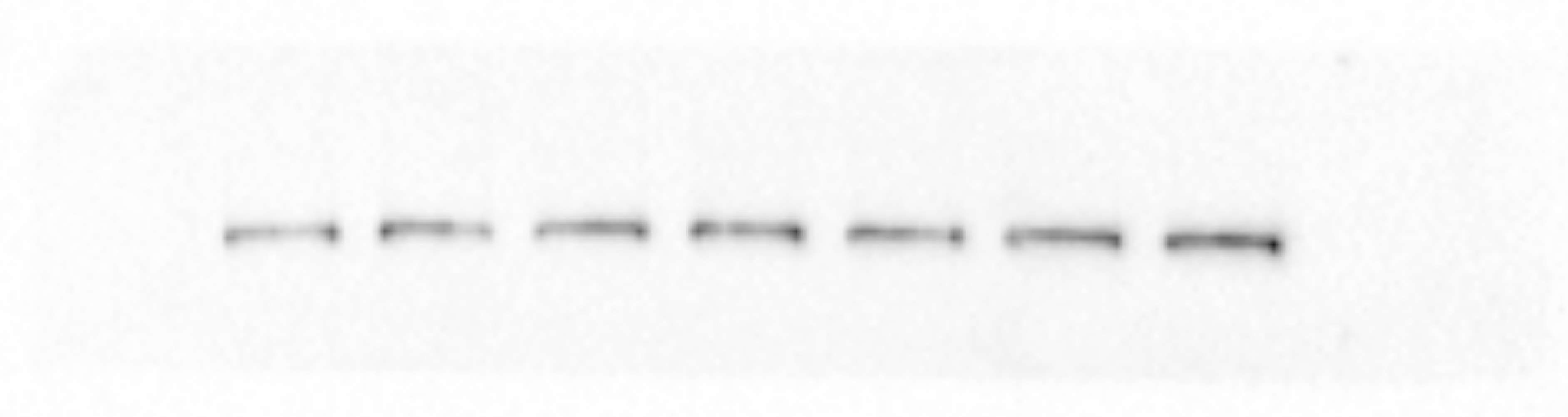

Supplement: Supplementary file 1 [file DataSheet_1.zip › Original image files/Fig 8E STAT5.jpg]

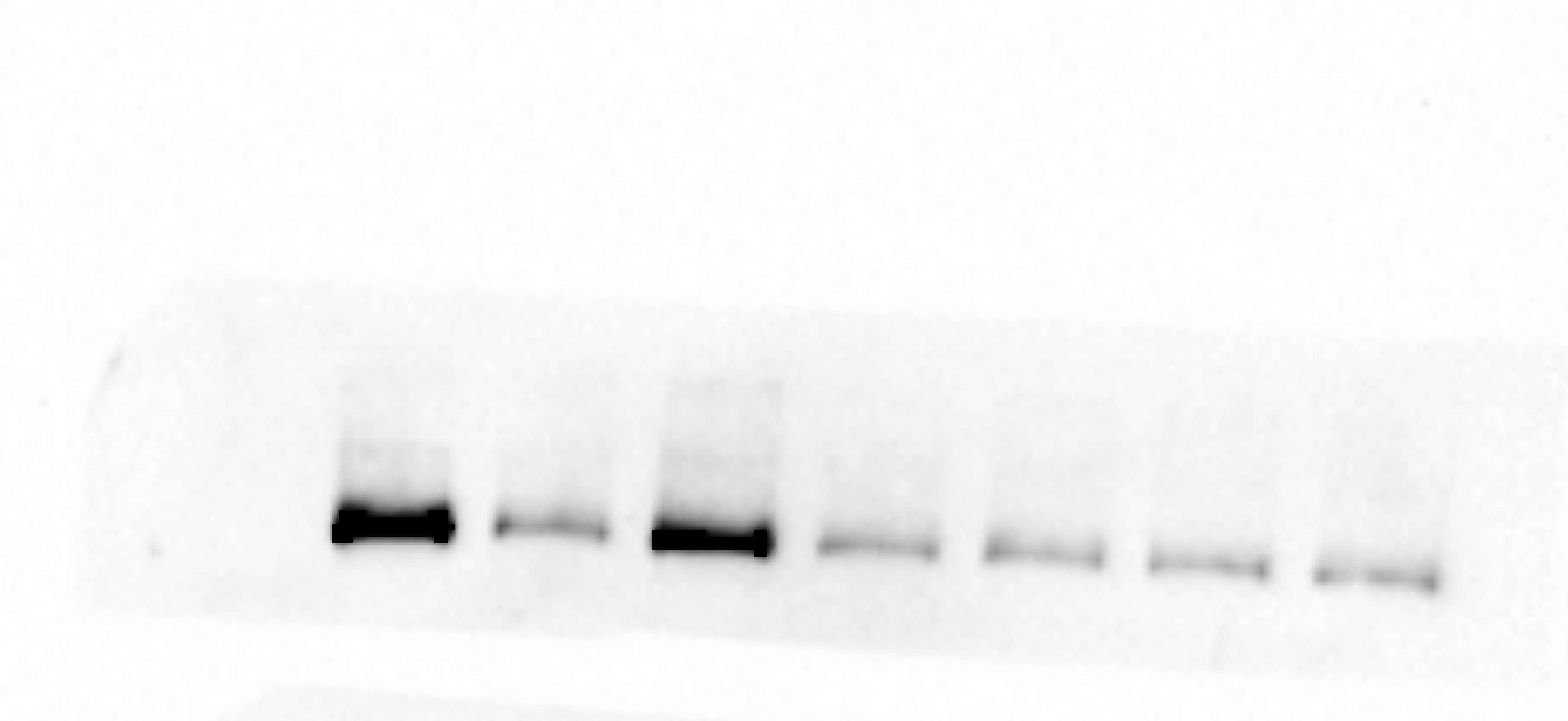

Supplement: Supplementary file 1 [file DataSheet_1.zip › Original image files/Fig 8E p-STAT5.jpg]

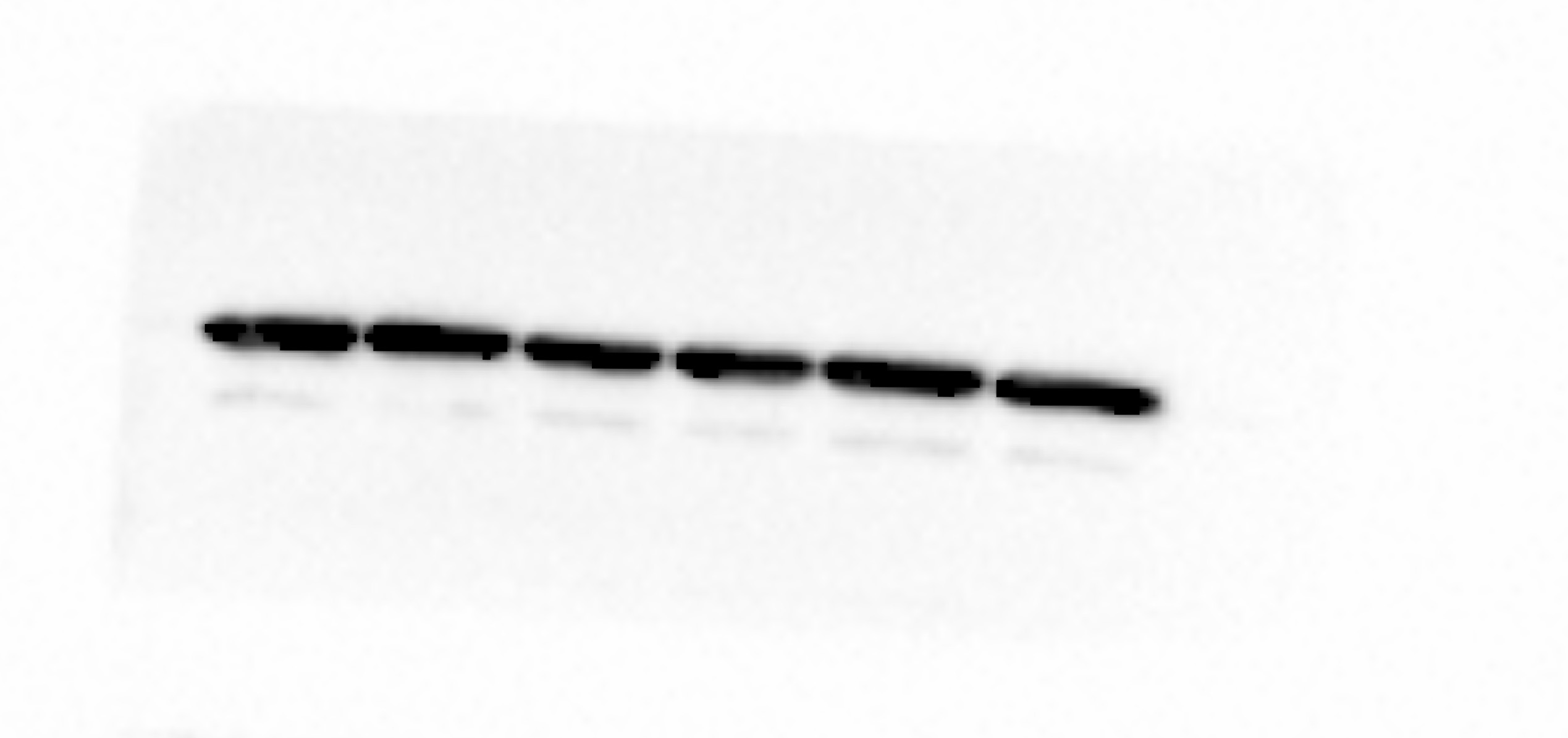

Supplement: Supplementary file 1 [file DataSheet_1.zip › Original image files/Figure 3G GAPDH.jpg]

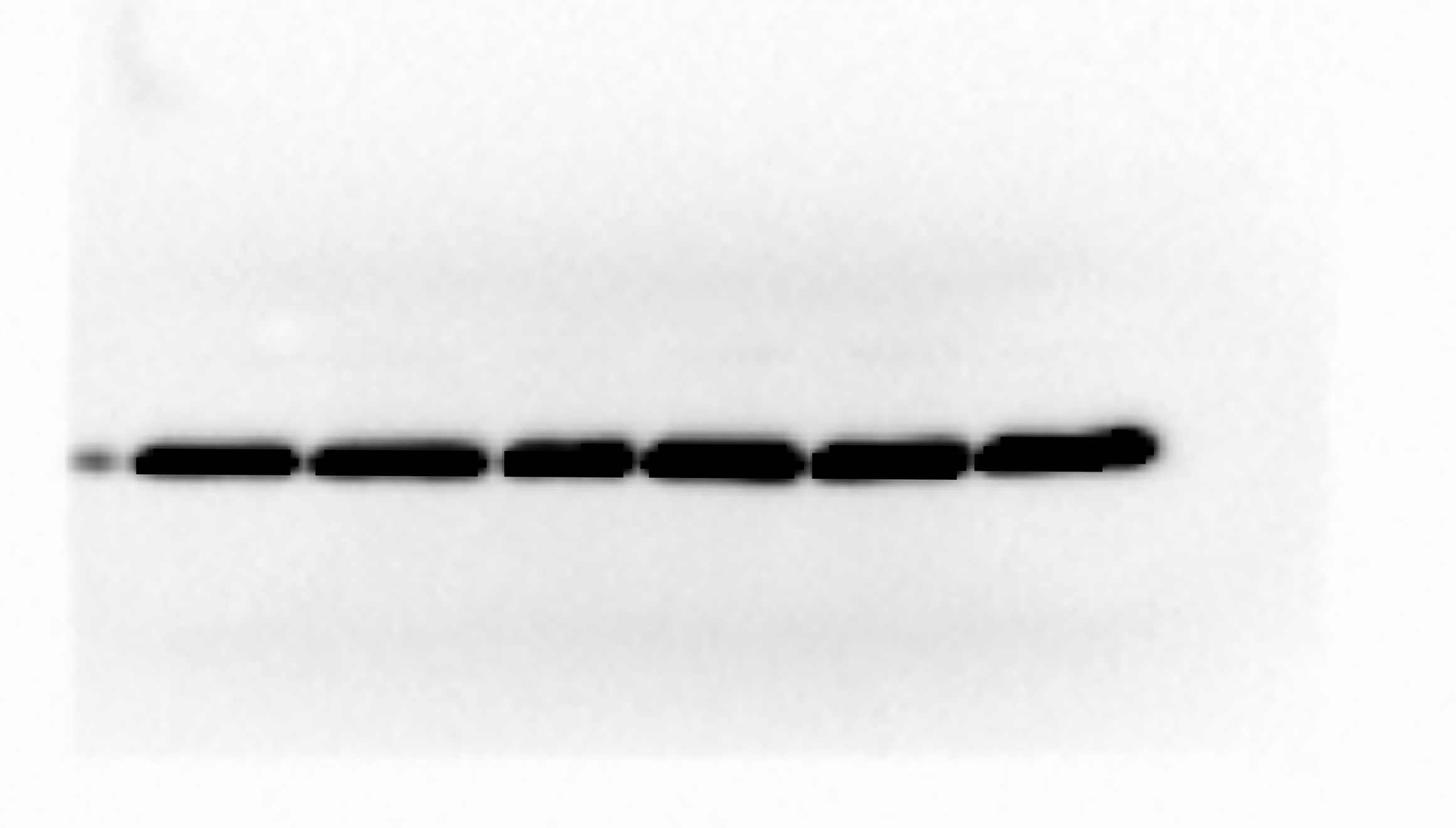

Supplement: Supplementary file 1 [file DataSheet_1.zip › Original image files/Figure 3G Stat3.jpg]

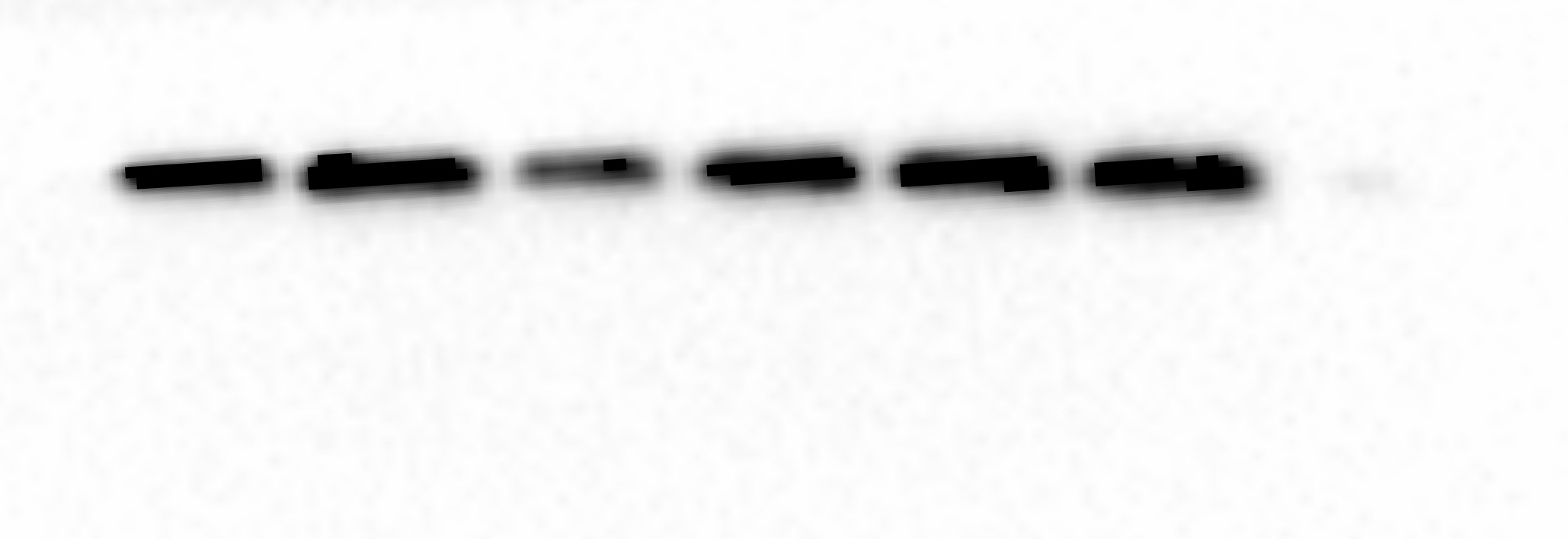

Supplement: Supplementary file 1 [file DataSheet_1.zip › Original image files/Figure 3G p-STAT3.jpg]

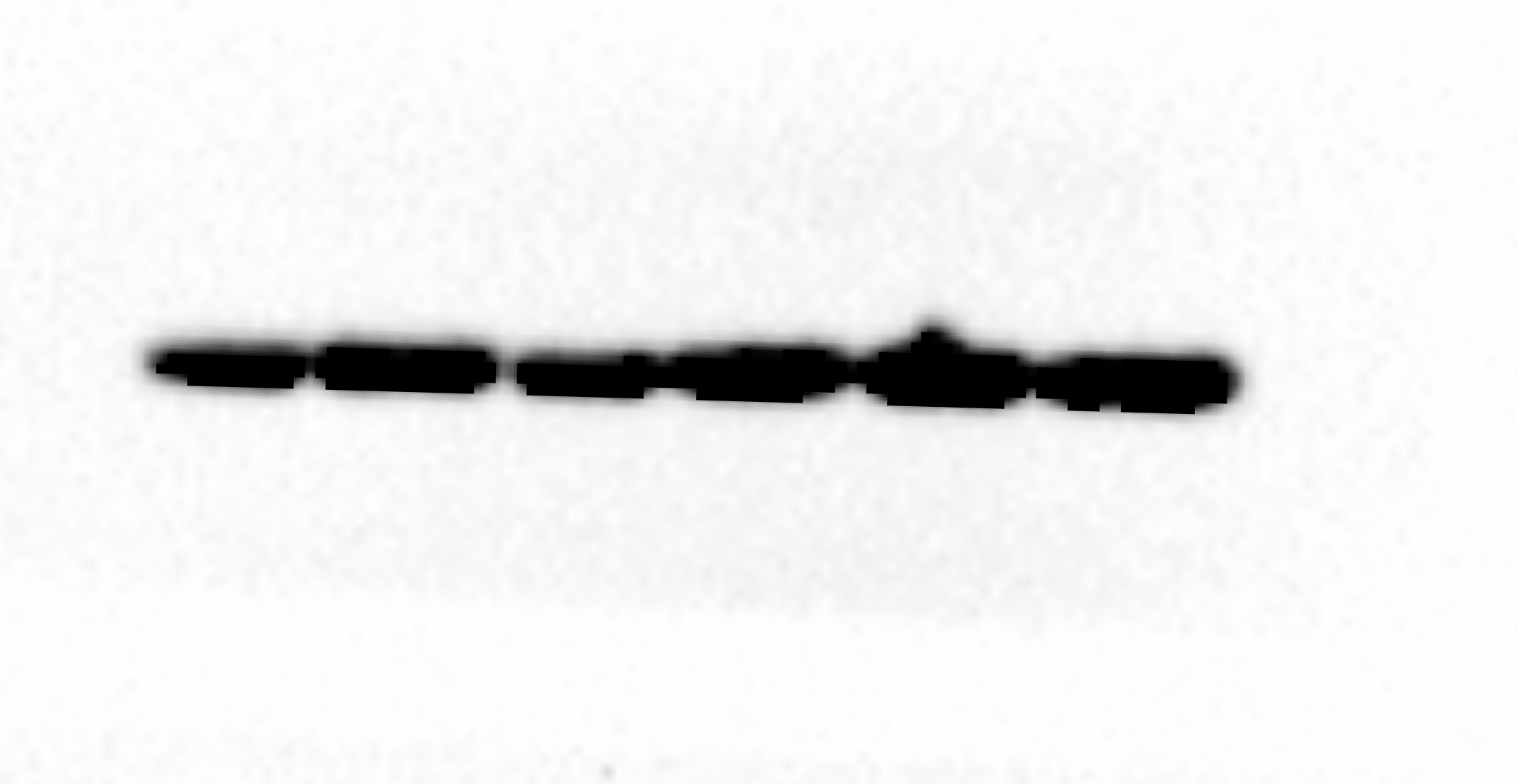

Supplement: Supplementary file 1 [file DataSheet_1.zip › Original image files/Figure 3H GAPDH.jpg]

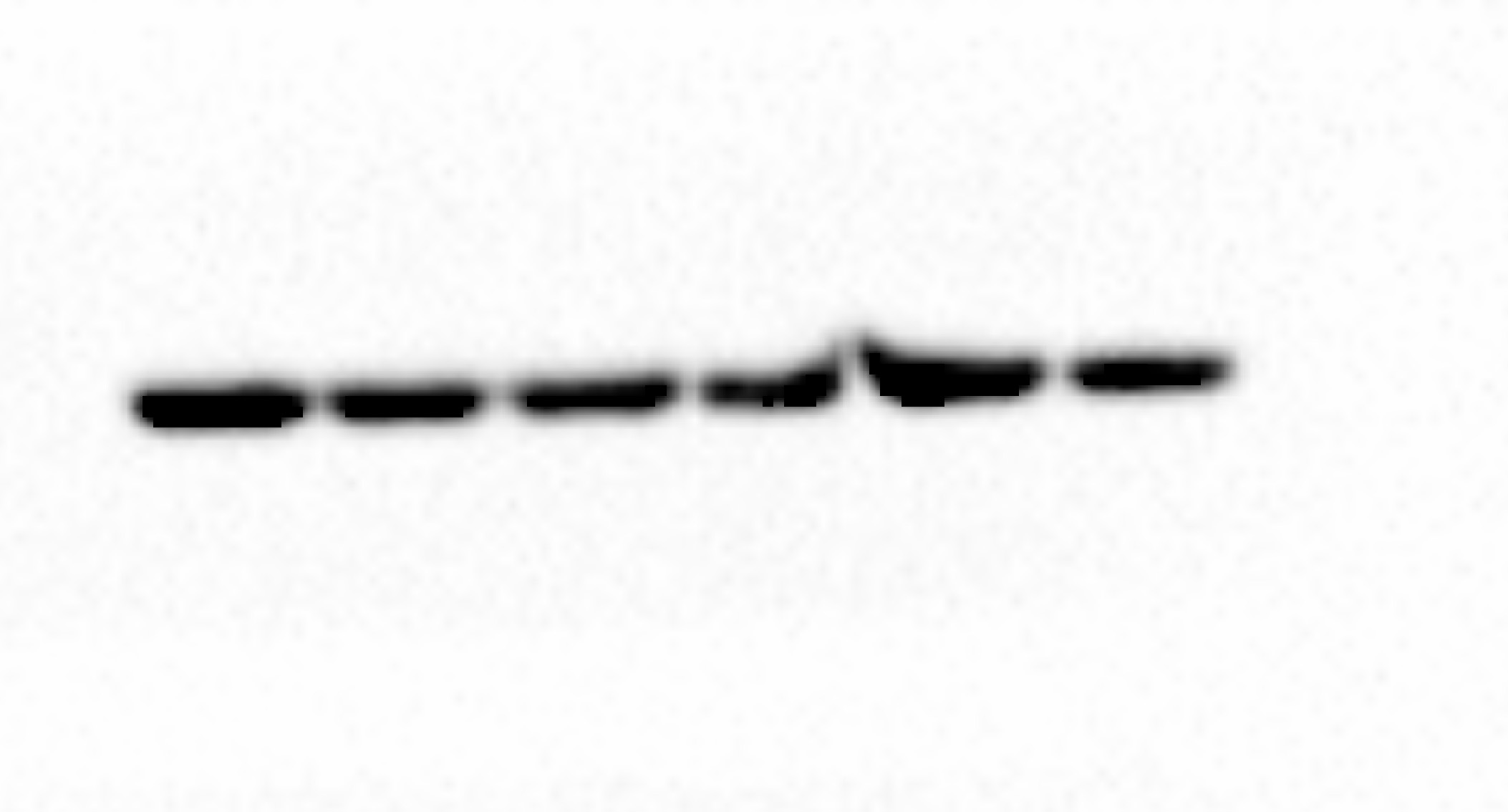

Supplement: Supplementary file 1 [file DataSheet_1.zip › Original image files/Figure 3H STAT5.jpg]

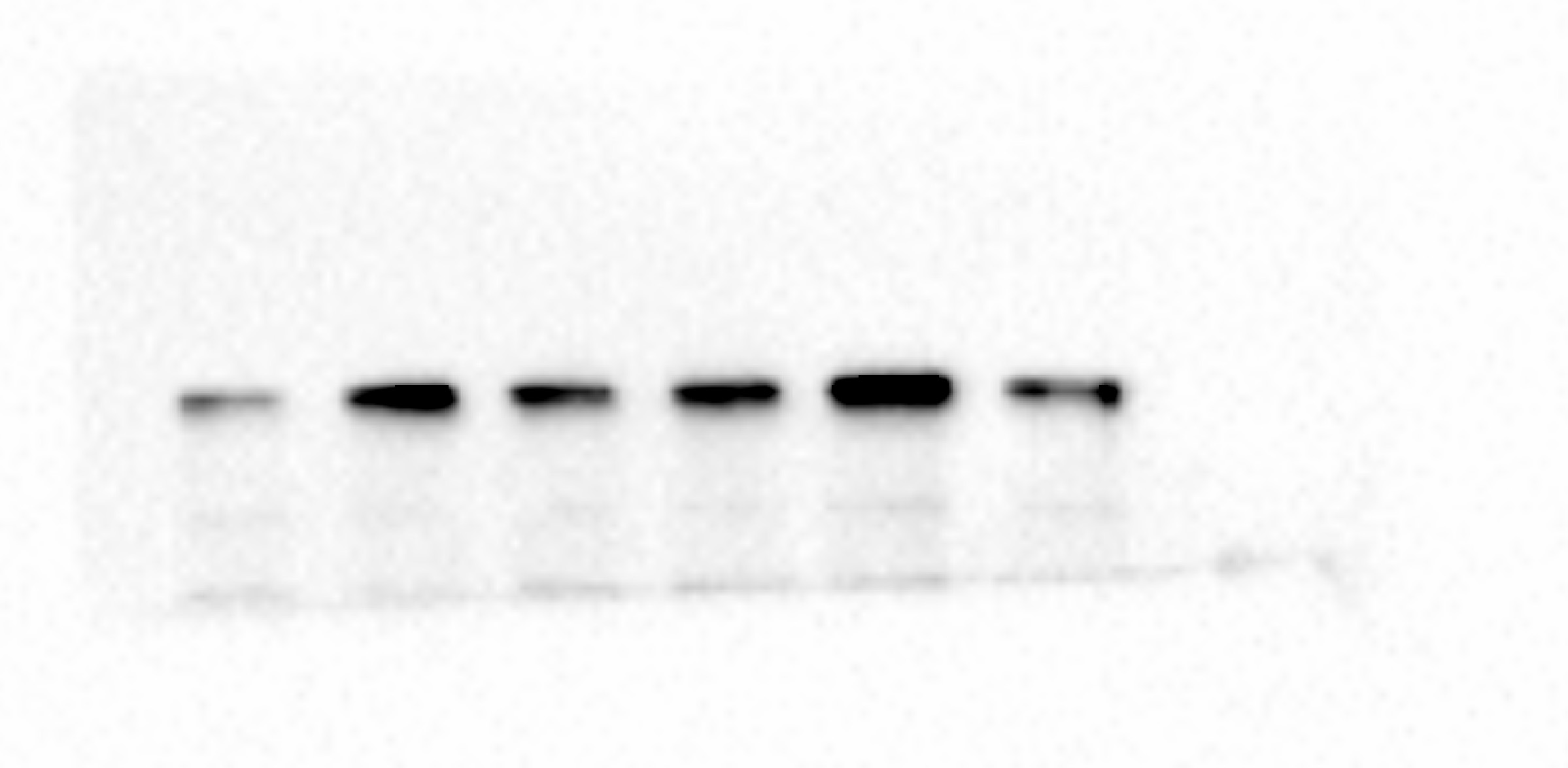

Supplement: Supplementary file 1 [file DataSheet_1.zip › Original image files/Figure 3H p-STAT5.jpg]

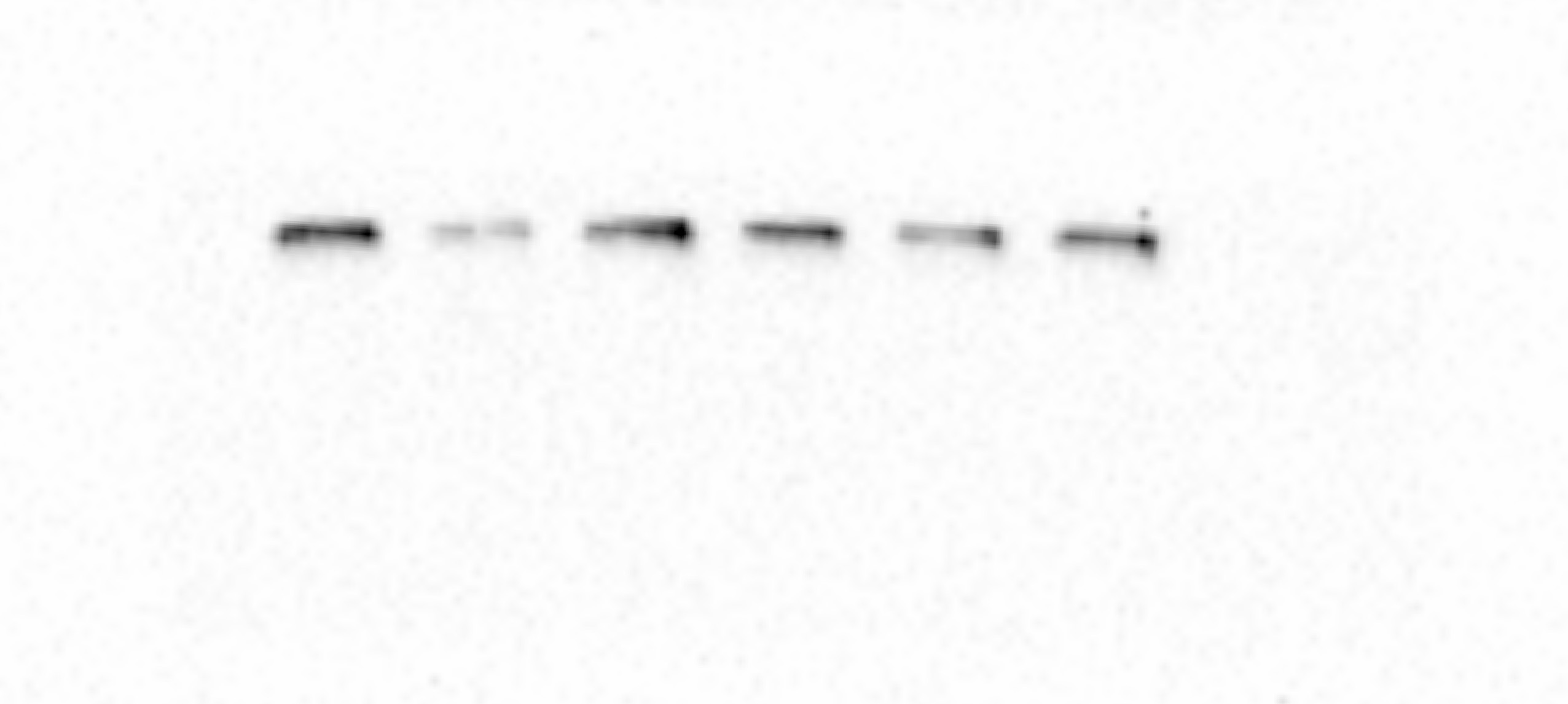

Supplement: Supplementary file 1 [file DataSheet_1.zip › Original image files/Figure 7E Foxp3.jpg]

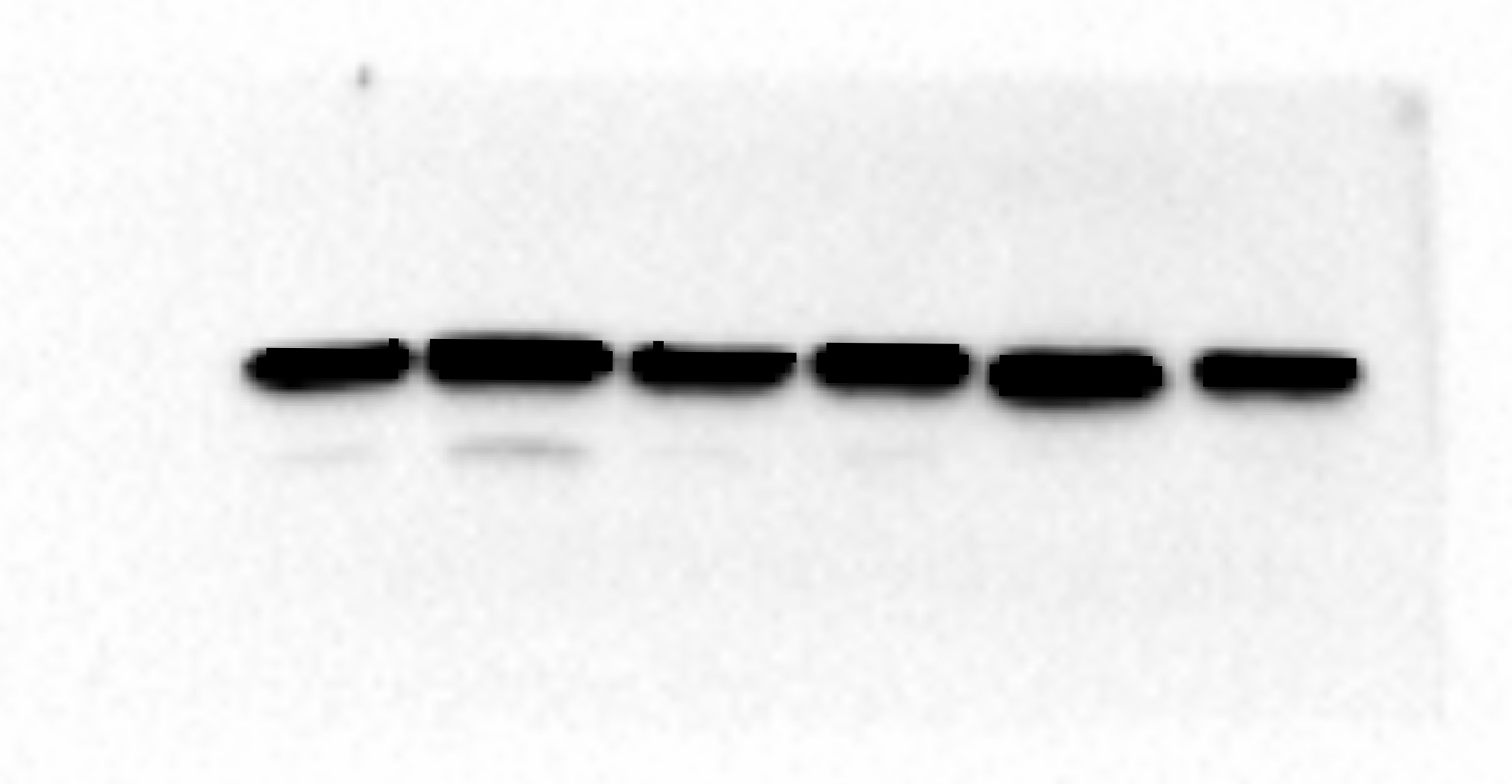

Supplement: Supplementary file 1 [file DataSheet_1.zip › Original image files/Figure 7E GAPDH.jpg]

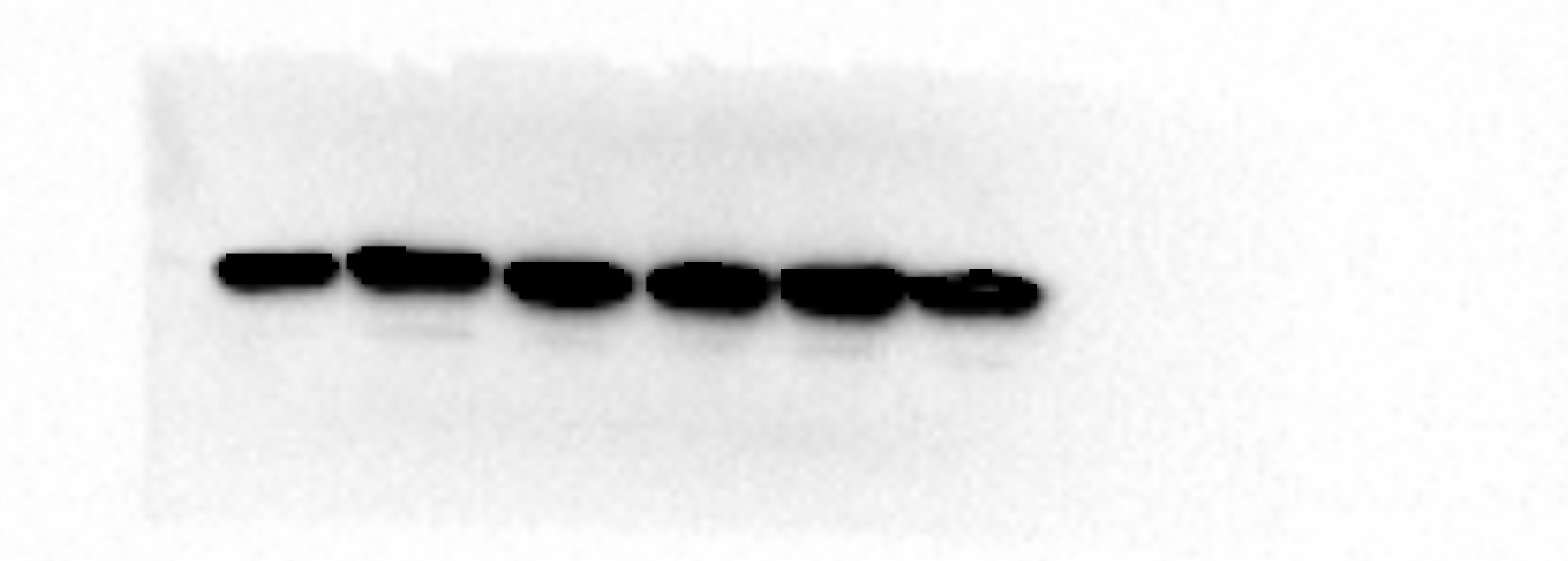

Supplement: Supplementary file 1 [file DataSheet_1.zip › Original image files/Figure 7F GAPDH.jpg]

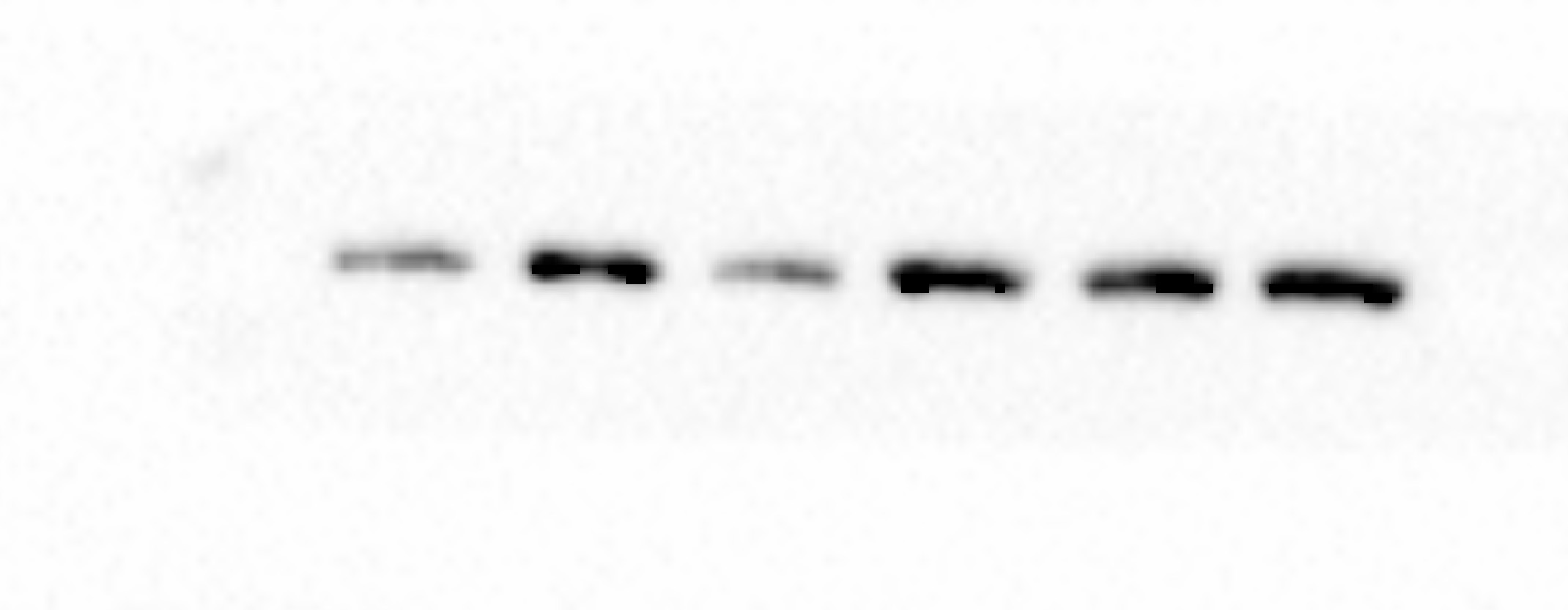

Supplement: Supplementary file 1 [file DataSheet_1.zip › Original image files/Figure 7F RORrt.jpg]

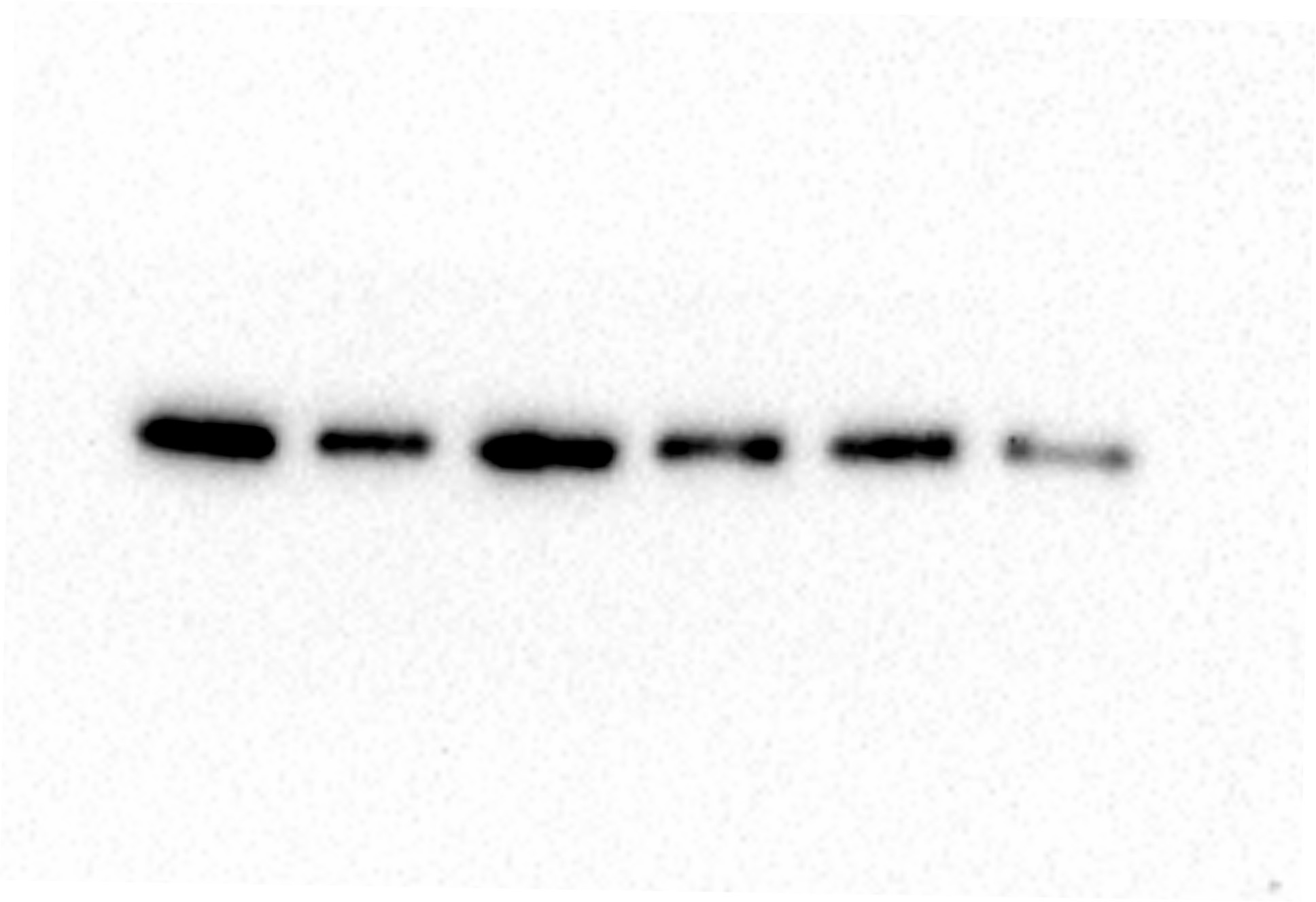

Supplement: Supplementary file 1 [file DataSheet_1.zip › Original image files/Figure 8C A2aR.jpg]

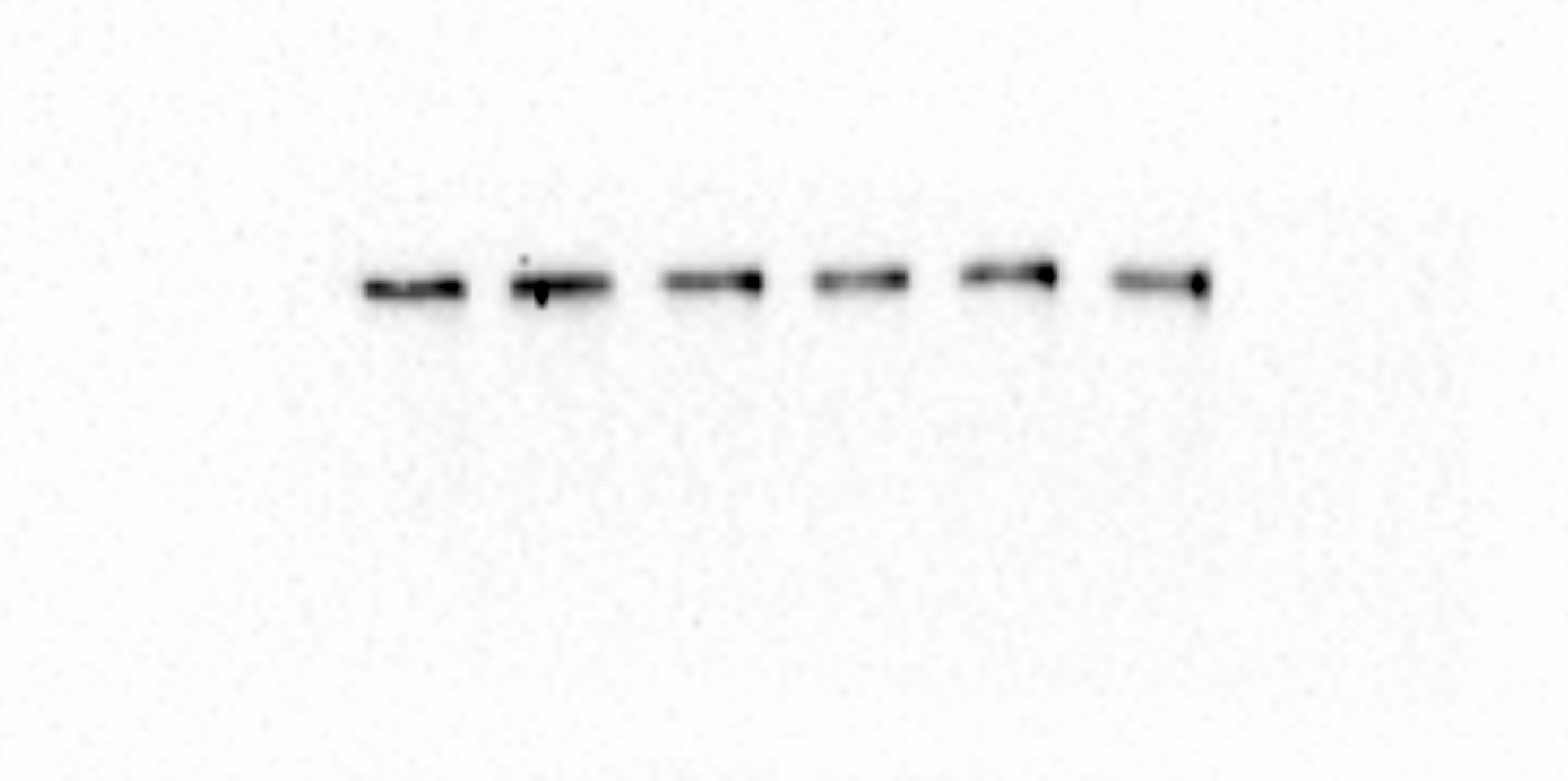

Supplement: Supplementary file 1 [file DataSheet_1.zip › Original image files/Figure 8C CREB.jpg]

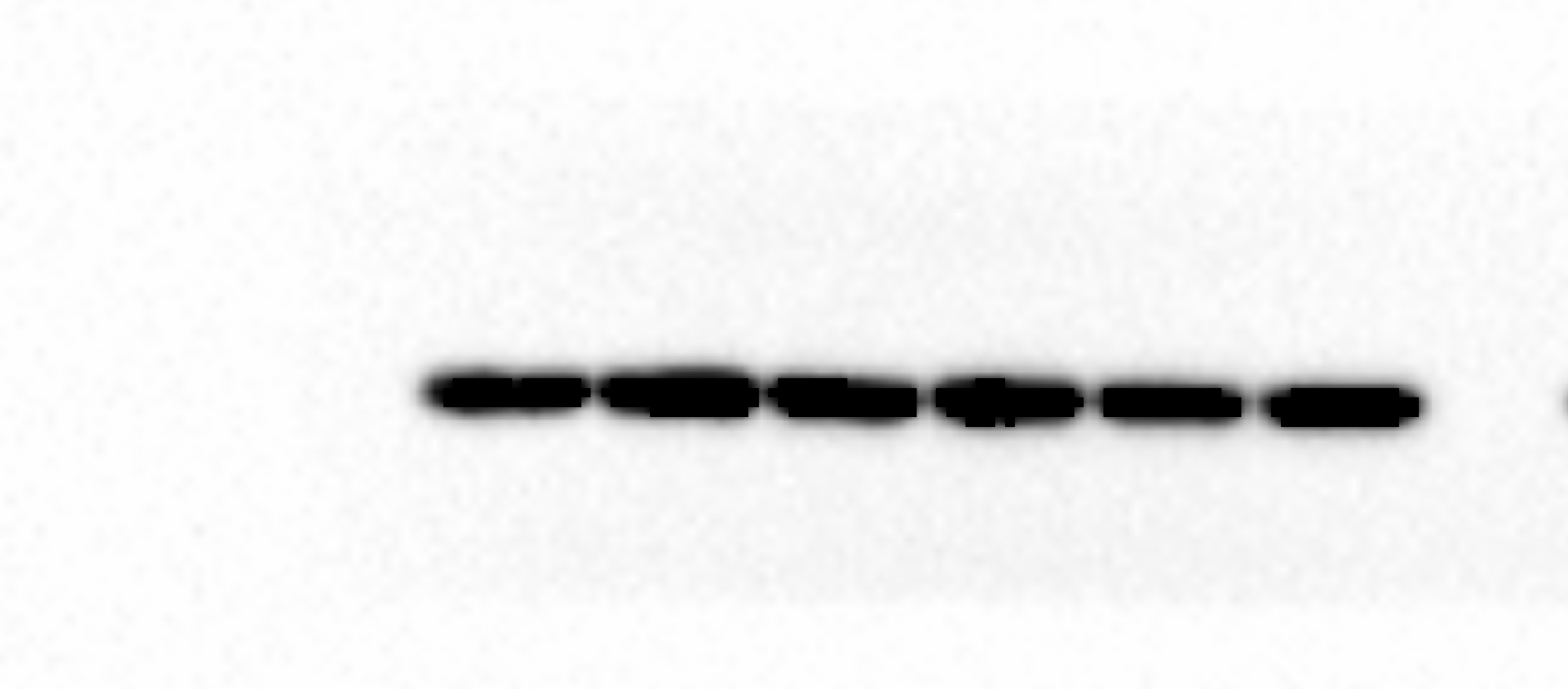

Supplement: Supplementary file 1 [file DataSheet_1.zip › Original image files/Figure 8C GAPDH.jpg]

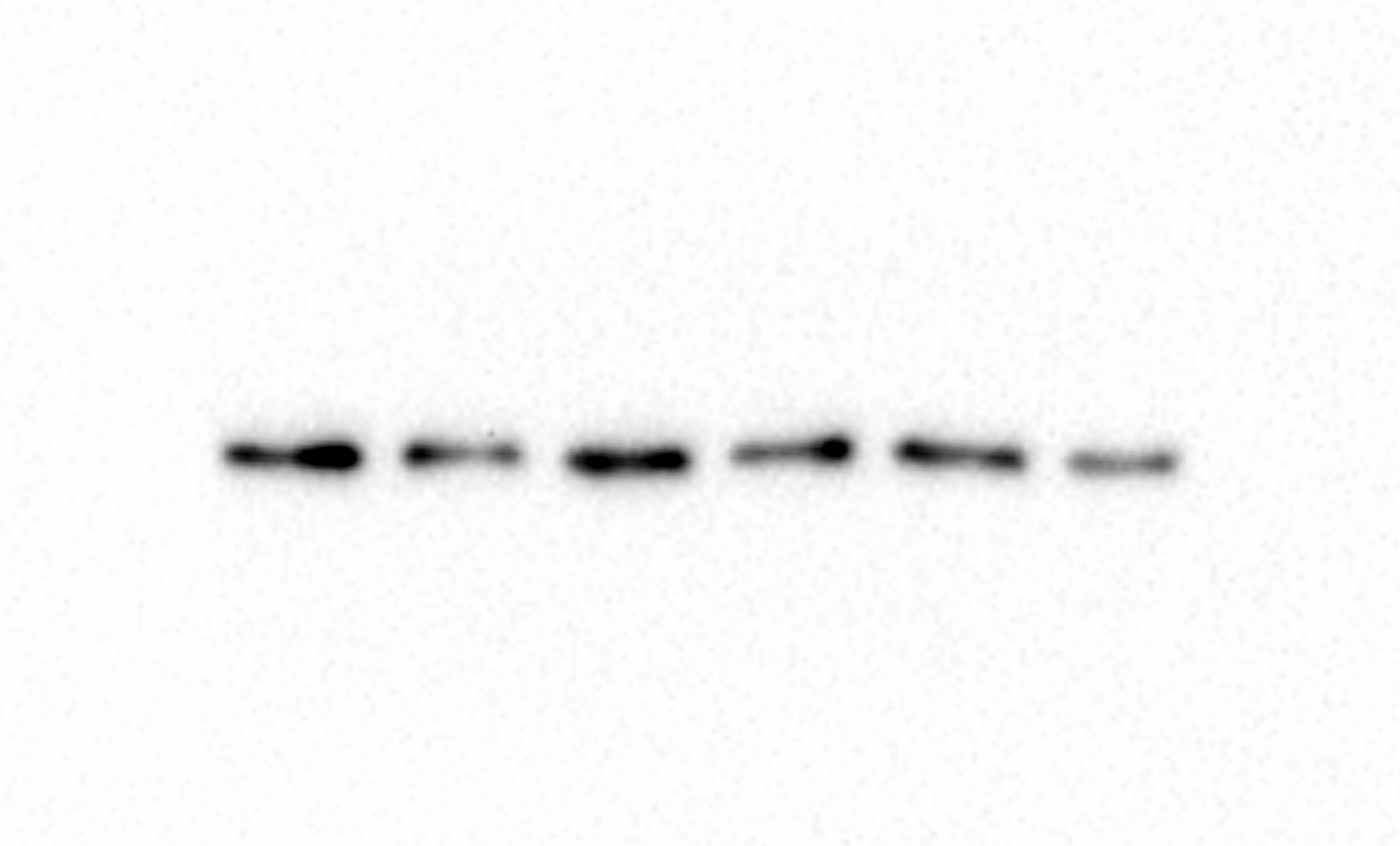

Supplement: Supplementary file 1 [file DataSheet_1.zip › Original image files/Figure 8C PKA.jpg]

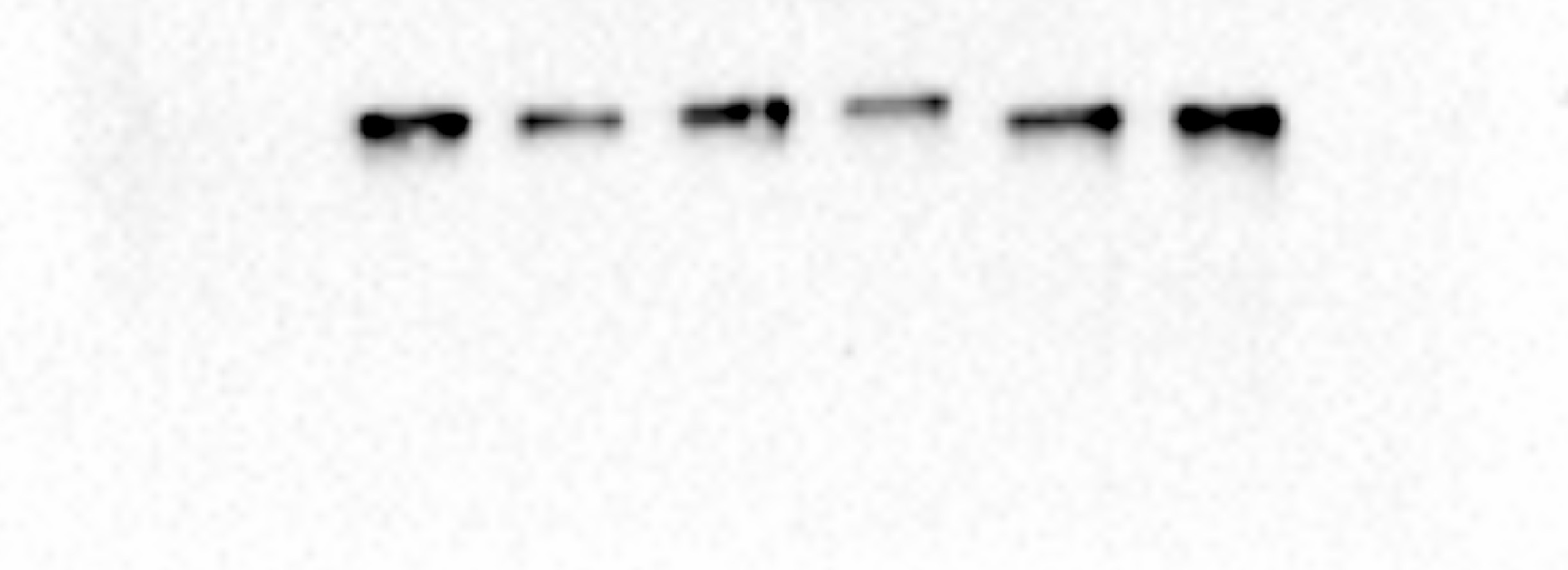

Supplement: Supplementary file 1 [file DataSheet_1.zip › Original image files/Figure 8C p-CREB.jpg]

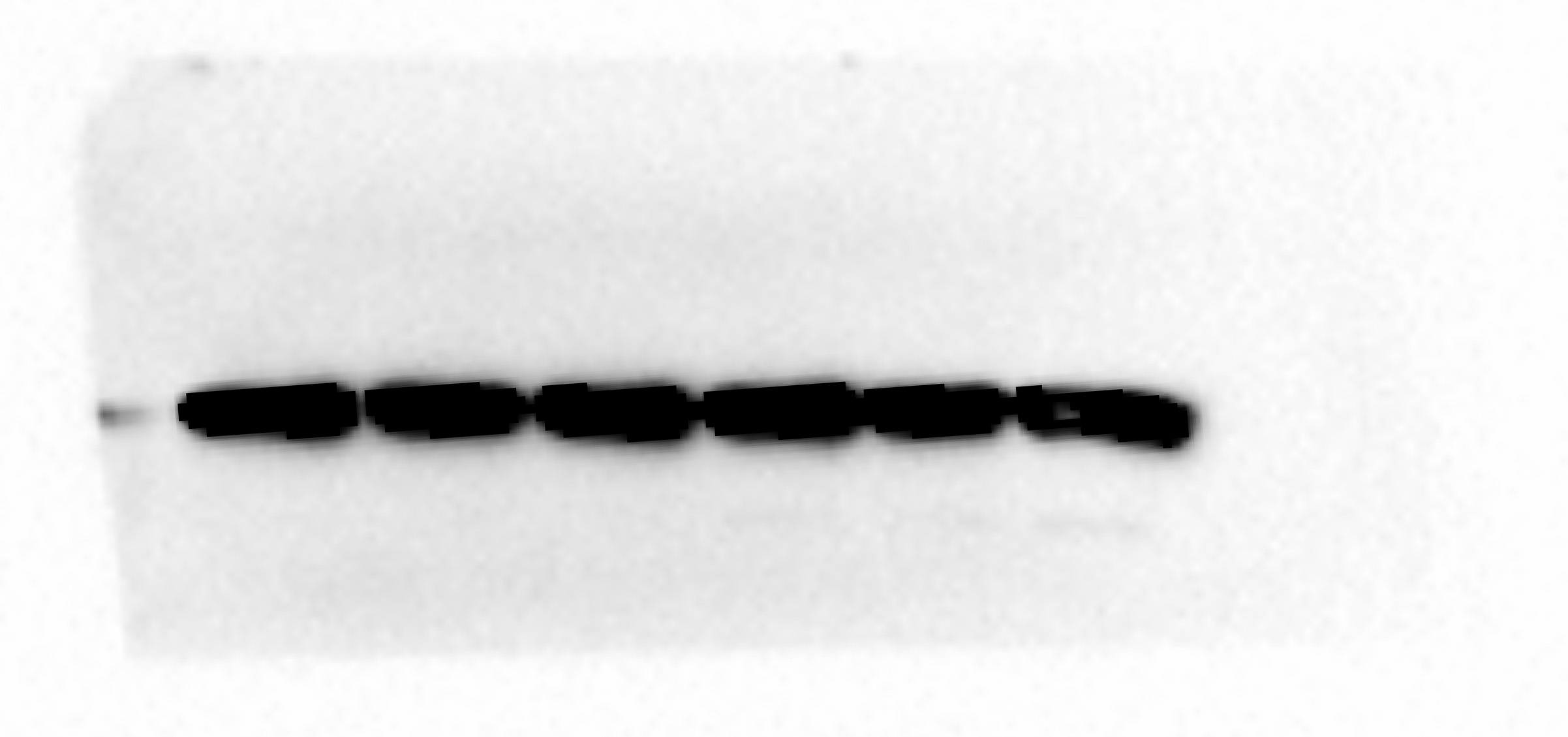

Supplement: Supplementary file 1 [file DataSheet_1.zip › Original image files/Figure 8F GAPDH.jpg]

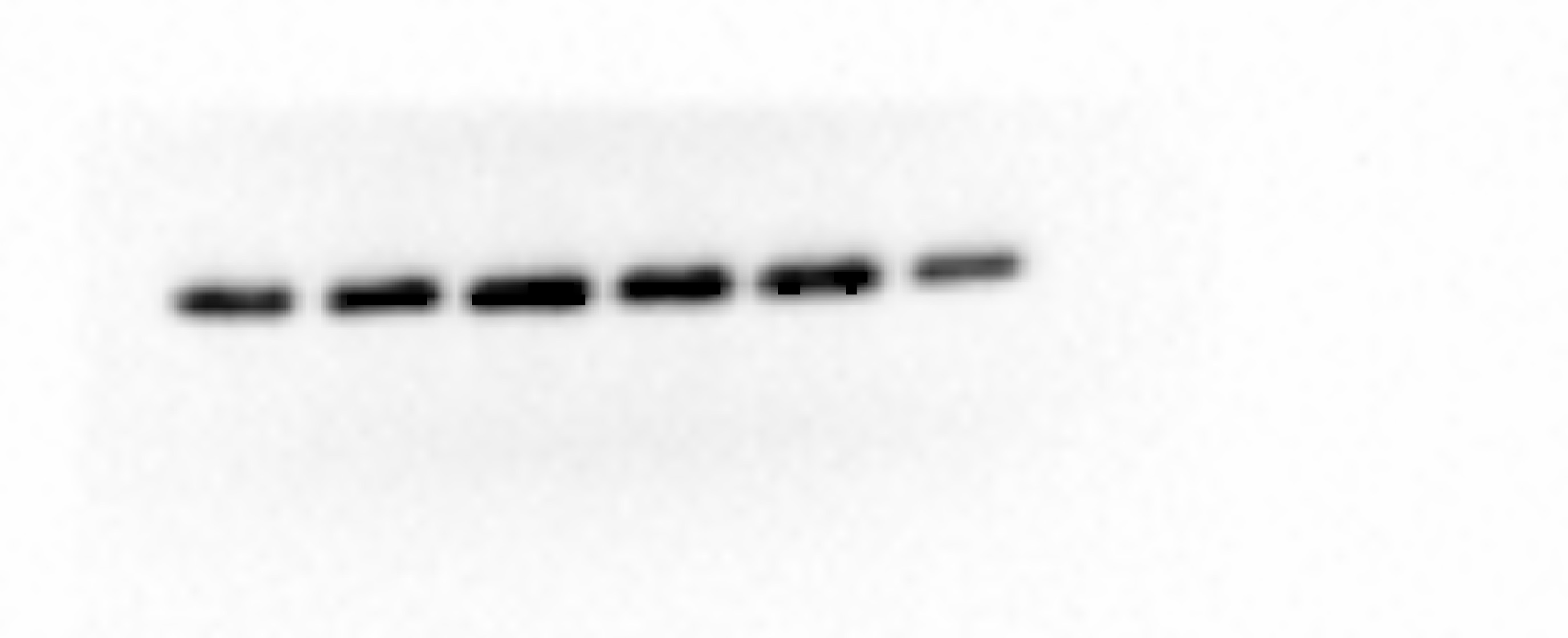

Supplement: Supplementary file 1 [file DataSheet_1.zip › Original image files/Figure 8F STAT3.jpg]

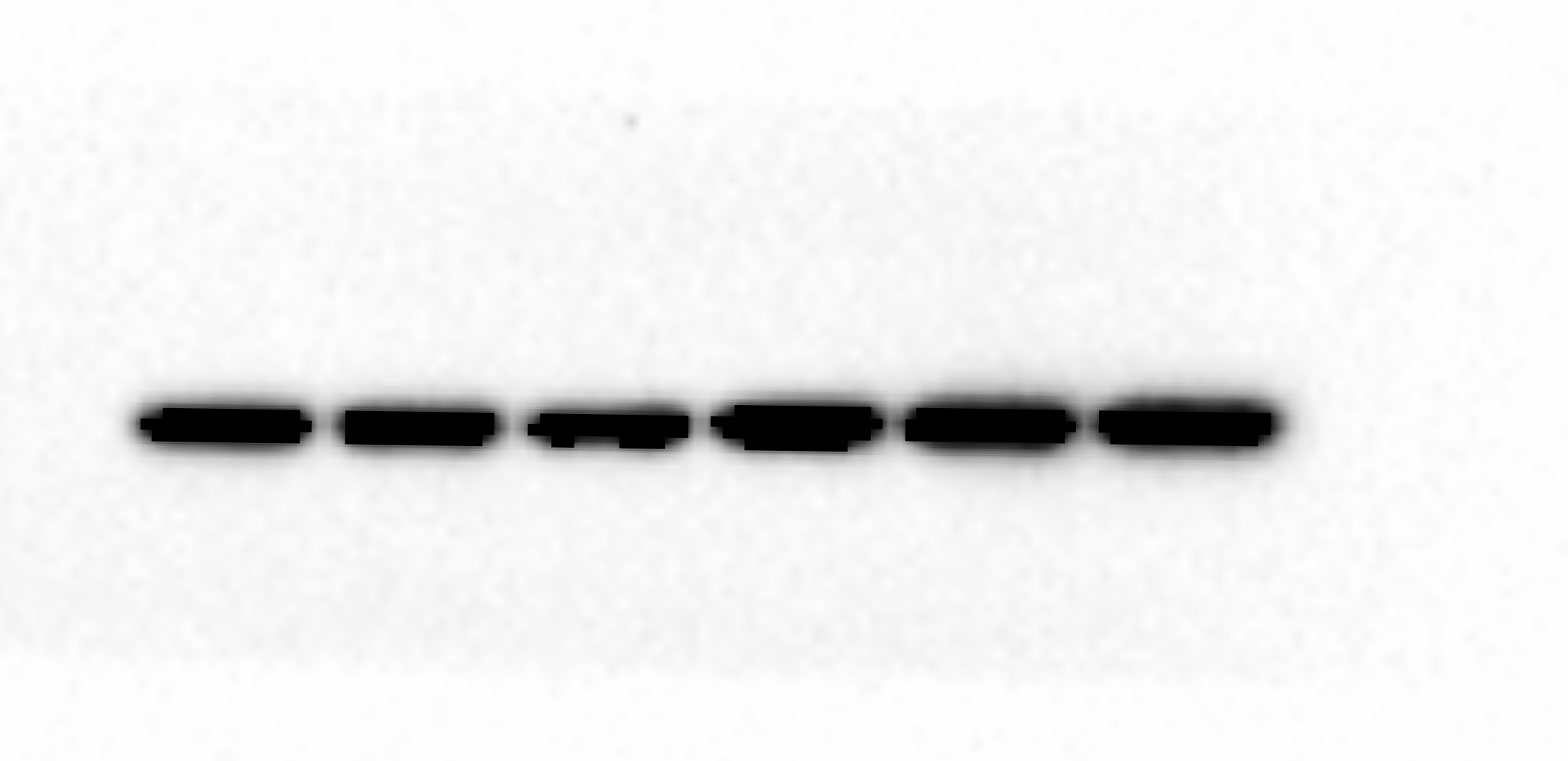

Supplement: Supplementary file 1 [file DataSheet_1.zip › Original image files/Figure 8F STAT5.jpg]

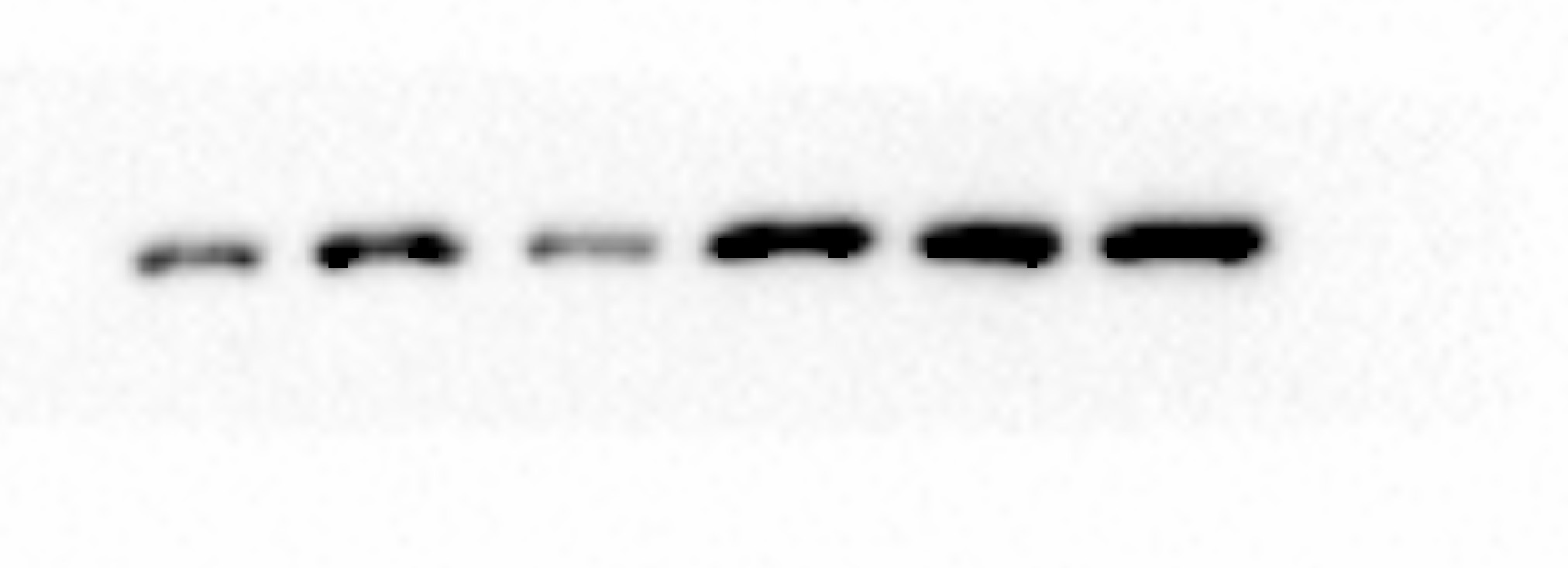

Supplement: Supplementary file 1 [file DataSheet_1.zip › Original image files/Figure 8F p-STAT3.jpg]

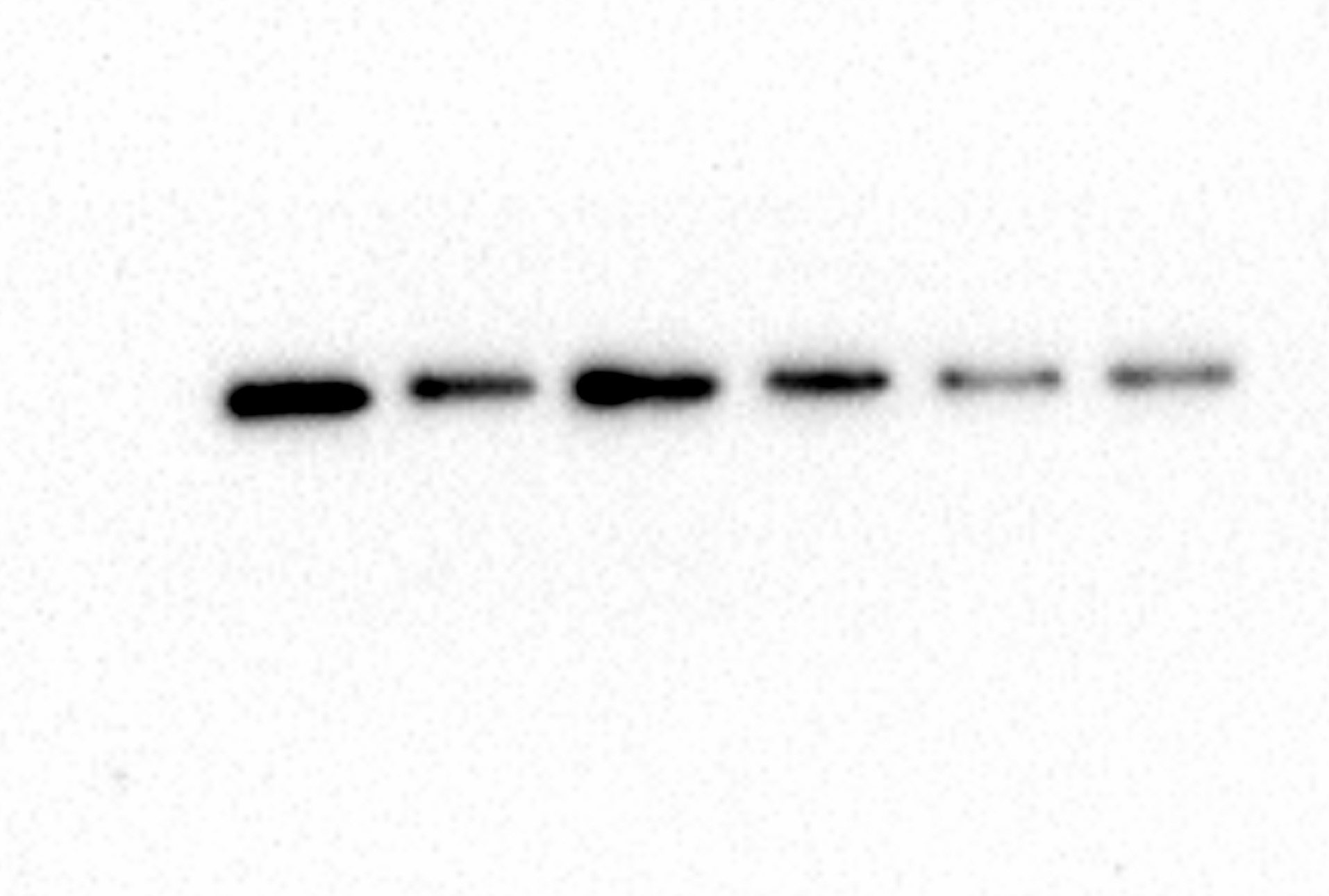

Supplement: Supplementary file 1 [file DataSheet_1.zip › Original image files/Figure 8F p-STAT5.jpg]
